# Real-world safety of tirofiban: a disproportionality analysis using data from FAERS and WHO-VigiAccess

**DOI:** 10.3389/fphar.2025.1713141

**Published:** 2025-12-11

**Authors:** Jia Li, Fang Wang, Lingquan Zhong, Lei Zhang, Kaiyun Ji, Yifan Zheng

**Affiliations:** 1 Department of Pharmacy, Guangxi Hospital Division of The First Affiliated Hospital, Sun Yat-sen University, Nanning, China; 2 Department of Pharmacy, The First Affiliated Hospital of Sun Yat-sen University, Guangzhou, China; 3 Department of Pharmacy, Guizhou Hospital of The First Affiliated Hospital of Sun Yat-sen University (The Affiliated Hospital of Guizhou Medical University), Guiyang, China; 4 Department of Pharmacy, Shanxi Provincial Integrated TCM And WM Hospital, Taiyuan, China; 5 Department of Pharmacy, Jincheng General Hospital, Jincheng, China; 6 Department of Clinical Pharmacy Translational Science, University of Michigan College of Pharmacy, Ann Arbor, MI, United States

**Keywords:** tirofiban, adverse events (AEs), FAERS database, WHO-VigiAccess database, disproportionality analysis

## Abstract

**Objective:**

This study aimed to detect tirofiban-related adverse event (AE) signals using the FAERS and WHO-VigiAccess databases to support safer clinical use.

**Methods:**

All tirofiban-related AE reports were retrieved from FAERS (2004Q1 to 2024Q4) and WHO-VigiAccess (Retrieval date 2024.12.15). Disproportionality analyses were performed using ROR (Reporting Odds Ratio), PRR (Proportional Reporting Ratio), BCPNN (Bayesian Confidence Propagation Neural Network), and MGPS (Multi-item Gamma Poisson Shrinker) to detect potential drug-AE associations. Time - to - onset was assessed with Weibull distribution and Kaplan-Meier methods. Sensitivity analyses were performed according to reporter type, age group, and sex to assess the robustness of the findings.

**Results:**

A total of 2,421 reports from FAERS and 3,485 from WHO-VigiAccess were identified. Bleeding and thrombocytopenia were the most frequent AEs, consistent with drug labeling. Notably, 21 AE signals suggestive of possible associations not listed in the current drug label were observed, such as vascular stent thrombosis and cardiogenic death, which require further studies to verify their causal relationship with tirofiban. The mean onset time was 12 h, and 96.49% occurred within 1 month of exposure. Subgroup analyses showed that male patients exhibited a stronger signal for thrombocytopenia, whereas female patients had a higher risk of ischemic heart disease. Elderly patients (≥65 years) more frequently experienced hemoglobin decreased, while younger patients (<65years) had a higher risk of thrombosis in device.

**Conclusion:**

This study identified both known and potentially novel tirofiban-related AEs. The rapid onset, particularly of bleeding and thrombocytopenia, highlights the importance of early monitoring. Management strategies, such as dose adjustment, temporary discontinuation, or supportive treatment including platelet transfusion, may help mitigate severe complications. These findings provide real-world evidence to guide safer tirofiban use, although further studies are required to confirm causality.

## 1 Introduction

Tirofiban is a highly selective, short half-life, rapid-acting platelet surface glycoprotein IIb/IIIa (GPIIb/IIIa) receptor antagonist (Yang et al., 2019), which has played an important role in the treatment of cardiovascular diseases since it was approved for marketing by the U.S. Food and Drug Administration (FDA) in 1998 (Lazarovici et al., 2019). It mainly blocks the binding sites on platelets and fibrinogen by binding to the GPIIb/IIIa receptor on the platelet surface, thus effectively inhibiting platelet aggregation and reducing the incidence of acute thrombus reocclusion (Topol et al., 1999; Li et al., 2024; Dobrzycki et al., 2007). Currently, tirofiban is widely used in the treatment of acute angina or acute myocardial infarction to prevent the occurrence of cardiac ischemic events; or as a prophylactic drug for intracoronary plaque excision or coronary angioplasty in patients with acute coronary syndromes, to reduce the occurrence of cardiac ischemic complications associated with sudden occlusion of coronary arteries (Li et al., 2024; Dobrzycki et al., 2007; Zhou et al., 2017; Cannon et al., 2001; Zhao et al., 1999).

In recent years, tirofiban has demonstrated significant efficacy in the treatment of acute ischemic stroke (Sun et al., 2021; Shi et al., 2023; Wang M. et al., 2024; Sang et al., 2023a; Zhao et al., 2024). Although the overall safety profile appeared favorable, the incidence of symptomatic intracranial hemorrhage was slightly higher in the tirofiban group in the RESCUE-BT2 study (1.0%) (Zi et al., 2023). Despite these therapeutic advantages, the clinical use of tirofiban is associated with potential safety concerns. Common AEs include bleeding and thrombocytopenia, with severe cases potentially leading to life-threatening complications such as alveolar hemorrhage and intracranial hemorrhage (Zi et al., 2023; Guo et al., 2012). The incidence of tirofiban-induced thrombocytopenia ranges from 0.4% to 5.6%, and in severe cases, it may increase mortality risk (Wang and Zou, 2023; Yi et al., 2018). As the use of tirofiban becomes increasingly widespread, it is crucial to raise awareness of its safety, especially regarding adverse events not explicitly mentioned in the drug labeling.

The Food and Drug Administration’s Adverse Event Reporting System (FAERS) is one of the largest databases for post-marketing safety surveillance, recording real-world data that can be analyzed to detect potential drug risk signals. Meanwhile, WHO-VigiAccess hosts the world’s most extensive adverse events (AEs) database, encompassing data from regular AE reports submitted by pharmacovigilance centers in over 150 countries. Recently, a pharmacovigilance study based on the FAERS database (Guo Q et al., 2025) analyzed AEs associated with tirofiban and identified several disproportionality signals suggesting potential safety concerns. However, that study relied solely on FAERS data up to Q3 2024, which may limit the generalizability of its findings. So our study will, for the first time, systematically and comprehensively mine AE signals for tirofiban using data from both FAERS and WHO-VigiAccess. To the best of our knowledge, it could enhance the robustness of signal detection by integrating two independent international pharmacovigilance databases. The inclusion of WHO-VigiAccess allows cross-validation across different reporting systems, thereby reducing database-specific bias and improving the reliability of detected signals. Through this dual-database approach, we extend data coverage to Q4 2024 and identify additional potential safety signals not previously reported, offering a more comprehensive perspective on tirofiban’s post-marketing safety profile.

## 2 Methods

### 2.1 Data sources and data processing

The FAERS database is a globally recognized repository for AE reporting, mainly collecting AE data submitted by drug manufacturers, healthcare professionals, and consumers (Dhodapkar et al., 2022). FAERS data has been accessible to the public since the first quarter of 2004, and is updated and released quarterly in both ASCII and XML formats for download. For this study, the original ASCII packet was downloaded for data mining and statistical analysis, at https://fis.fda.gov/extensions/FPD-QDE-FAERS/FPD-QDE-FAERS.html. A total of 22,375,298 cases were initially obtained. As the database relies on spontaneous reporting, which may include duplicate or withdrawn/deleted reports, this study adhered strictly to the data cleaning guidelines provided by the FDA.

The WHO Uppsala Monitoring Centre (UMC) is the WHO Collaborating Centre for International Drug Monitoring and has the world’s largest AE database. Since 1968, pharmacovigilance centers in more than 150 countries have partnered with UMC to regularly submit AE reporting data. In 2015, WHO launched VigiAccess to make AE reporting information from the VigiBase database available to the public at http://www.Vigiaccess.org/. The database is updated weekly. On December 15, 2024, we accessed the VigiAccess database to retrieve all publicly available drug data, which were subsequently exported into Excel for further analysis. A total of 24,152 drugs with baseline information and 24,127 drugs with AE information were obtained. The WHO information system collects data on age group, sex, year of reporting, and global continent.

AEs reported in both databases were coded using terminology from the Medical Dictionary of Regulatory Activities (MedDRA27.1). These coded terms were arranged in a hierarchy of 5 categories, both broad (system organ class [SOC]) and category-specific (e.g., preferred term [PT]).

### 2.2 Eligibility criteria

We obtained data from the FAERS database for a total of 84 quarters, ranging from the first quarter of 2004 (2004Q1) to the fourth quarter of 2024 (2024Q4). Additionally, on December 15, 2024, we accessed the WHO-VigiAccess database to retrieve the relevant AE report data. In these datasets, tirofiban was designated as a primary suspected (PS) drug. A Suspect product is defined as a drug or biologic that the reporter believes to be associated with the reported AE.

### 2.3 Statistical analysis

In this study, we employed a combination of disproportionality analysis methods to detect signals of AEs. The methods used include the Reporting Odds Ratio (ROR) (Sakaeda et al., 2013), Proportional Reporting Ratio (PRR) (Kelly et al., 2007), Bayesian Confidence Propagation Neural Network (BCPNN) (Bate et al., 1998), and Multi-item Gamma Poisson Shrinker (MGPS) (Szarfman et al., 2004). These methods are detailed in Supplementary Table S1. Both ROR and PRR are frequency-based methods known for their high sensitivity but lower specificity, while BCPNN and MGPS are Bayesian methods suitable for handling complex variables, though they tend to have lower sensitivity. Although there is no gold standard for signal detection methods, each approach has its own characteristics, with distinct advantages and limitations in terms of applicability and feasibility within databases. Therefore, We define a PT as a positive signal if it simultaneously meets the threshold criteria of all four methods and is present in both the FAERS and WHO-VigiAccess databases. Consistent detection of signals across multiple methods helps reduce the likelihood of false positives. The strength of the signal is indicated by the magnitude of the parameter values, and higher values suggest stronger signals. The specific formulas and criteria for these algorithms are provided in Supplementary Table S2. For statistical analysis, we used SAS 9.4 software and Excel 2019.

### 2.4 Time to onset analysis

To thoroughly evaluate the TTO characteristics, we utilized several statistical measures, including the median, interquartile range, minimum, maximum, and Weibull shape parameters (Kinoshita et al., 2020). The Kaplan-Meier method was used to illustrate the cumulative incidence of AEs associated with tirofiban (Wang Q. et al., 2024). Changes in the incidence of AE risk over time can be identified and predicted by the Weibull distribution test. This distribution is characterized by two parameters: the scale parameter (α), which determines the proportion or width of the distribution, and the shape parameter (β), which influences the curvature of the distribution. In our analysis of onset time, the shape parameter was crucial to predict the hazard of AEs over time, categorizing the outcome as early, random, or wear-out failure type. When β is less than 1 and its 95% confidence interval (CI) is also below 1, it suggests a decreasing risk of AEs over time, indicating an early failure pattern. Conversely, if β is approximately 1 and its 95% CI contains 1, the risk is estimated to remain constant over time, representing a random failure pattern. Finally, if β exceeds 1 and its 95% CI does not encompass 1, it implies an increasing risk over time, characteristic of a wear-out failure pattern (Sauzet et al., 2013; Liu et al., 2024).

### 2.5 Sensitivity analysis

To evaluate the robustness of the observed tirofiban-related AE signals, sensitivity analyses were performed stratified by reporter type, age, and sex. Disproportionality analyses (ROR, PRR, BCPNN, and MGPS) were conducted separately within subgroups of healthcare professionals and non-healthcare reporters to examine whether the reporting source influenced signal detection. For age- and gender-difference analyses, the association between AEs and demographic variables was assessed using the chi-square (χ^2^) test or Fisher’s exact test, depending on the data distribution and expected cell counts. A two-tailed P value <0.05 was considered statistically significant. All analyses were conducted using SAS 9.4. These analyses allowed us to determine whether the identified AE signals were consistent across different patient subgroups and reporting sources, enhancing the reliability and generalizability of the findings.

## 3 Result

### 3.1 General characteristics

**FIGURE 1:**
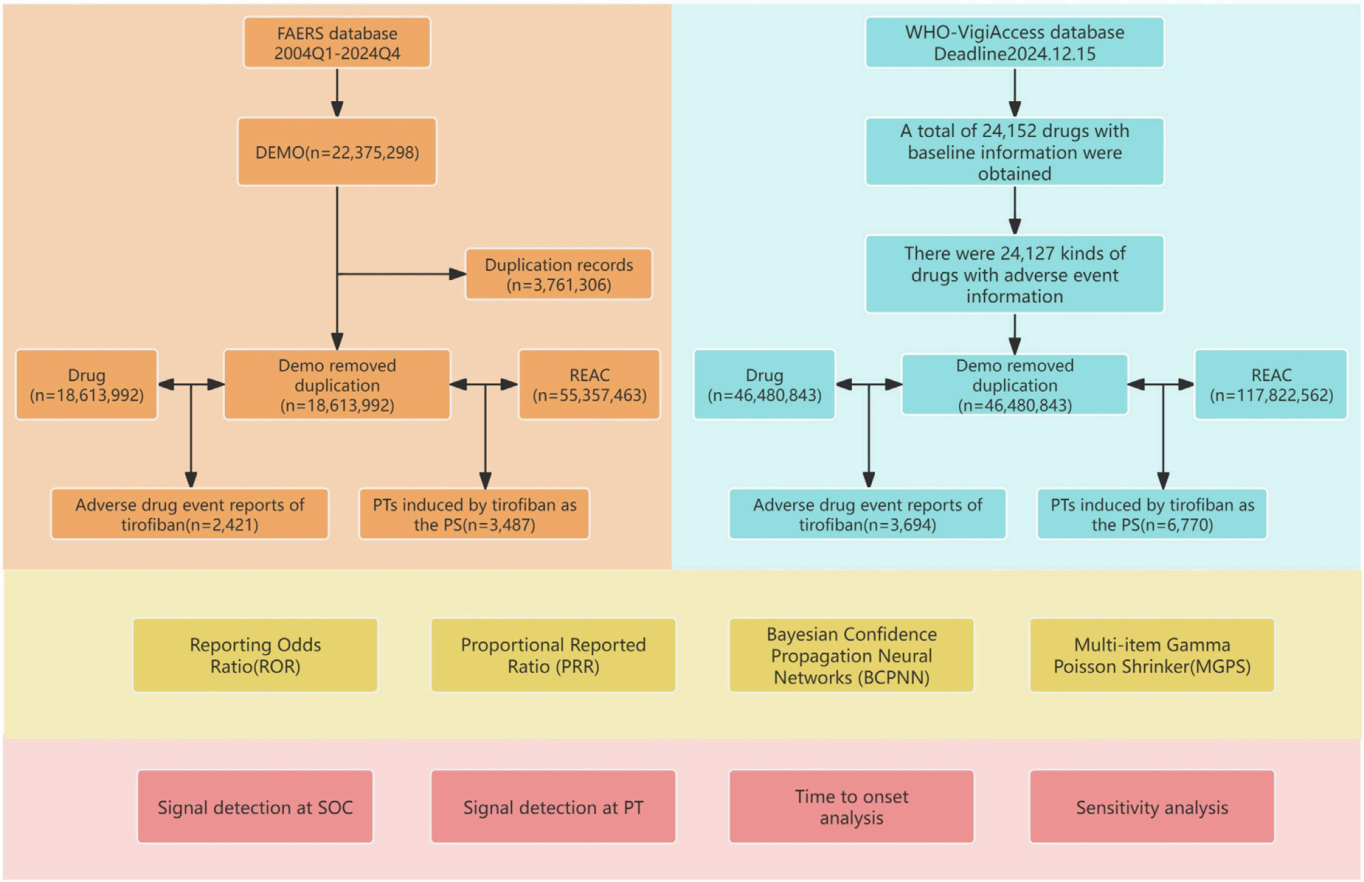
Flowchart of the study. The study consisted of data collection and cleaning, disproportionate analysis methods for calculating signal strength, and presentation of results.

In this study, a total of 55,357,463 AE reports involving 18,613,992 patients were obtained from the FAERS database after data cleaning and deduplication. Among these, 3,487 AE reports were reported with tirofiban as the primary suspected drug, involving 2,421 patients. Additionally, through the WHO-VigiAccess database, we extracted a total of 117,822,562 AE reports involving 46,480,843 patients, of which 6,770 AE reports were related to tirofiban, involving 3,694 patients. The detailed process of data collection, interpretation, and analysis is shown in Figure 1.

**FIGURE 2:**
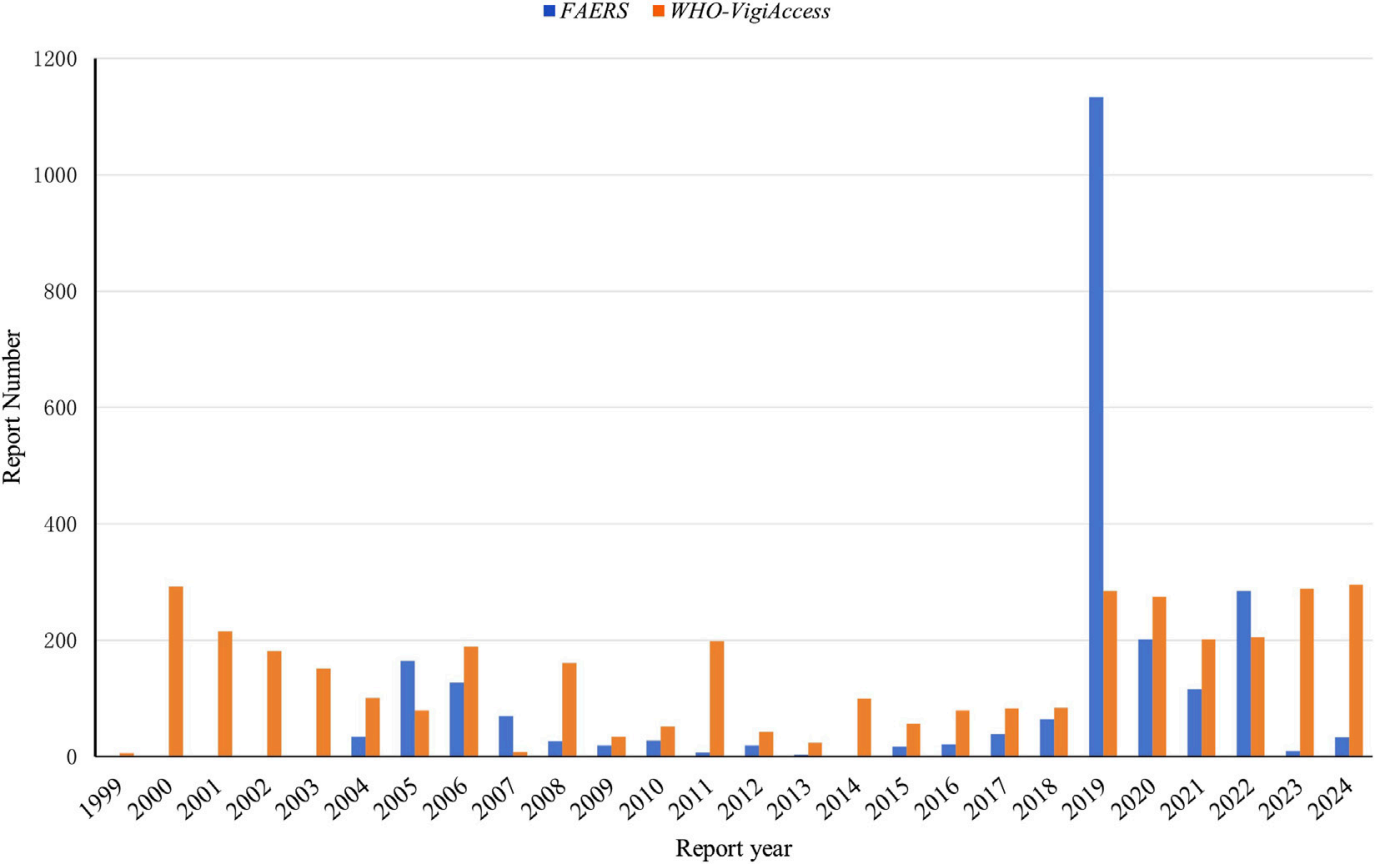
Annual distribution of AE reports involving tirofiban in the FAERS and WHO-VigiAccess databases.

The clinical characteristics of tirofiban-related AEs are summarized in Table 1. Excluding reports with missing demographic data, AEs were more frequently reported in older adults and males across both databases. In reports with clealy specified gender,15.49% of AEs in the FAERS occurred in males and 11.24% in females. A total of 73.28% of all reports lacked gender information and were therefore excluded from the gender-based distribution analysis. In the WHO-VigiAccess, 61.37% of AEs occurred in males and 28.64% in females, while 9.99% of the total reports lacked gender information. Notably, in WHO-VigiAccess, the male to female ratio was approximately 2:1. With respect to age distribution, the highest proportion of AEs was observed among patients aged ≥65 years (12.64% in FAERS, 47.03% in WHO-VigiAccess), followed by those aged 45–64 years (7.19% in FAERS, 32.89% in WHO-VigiAccess). Temporal analysis of AE reporting, as shown in Figure 2, indicates that the FAERS database recorded the highest number of reports in 2019 (n = 1,134, 46.84%). In contrast, AE reports in the WHO-VigiAccess database were primarily concentrated in two periods: 2000–2006 and 2019–2024.

**TABLE 1:** Characteristics of AE reports related to tirofiban in both the FAERS and WHO-VigiAccess databases.

Characteristics	*FAERS(n = 2,421)*		*WHO-VigiAccess(n = 3,694)*	
	*N*	*%*	*N*	*%*
Sex				
Female	272	11.24	1058	28.64
Male	375	15.49	2267	61.37
Unknown	1774	73.28	369	9.99
Age (years)				
<18	1	0.04	6	0.27
18–44	35	1.45	158	4.28
45–64	174	7.19	1215	32.89
≥65	306	12.64	1737	47.03
Unknown	1905	78.69	577	15.62
Continent				
Asia	1498	61.88	1511	40.9
Americas	190	7.85	1162	31.46
Europe	136	5.62	898	24.31
Africa	57	2.35	25	0.68
Oceania	14	0.58	98	2.65
Unknown	526	21.73	-	-

In the FAERS database, the majority of AE reports were submitted by healthcare professionals, including physicians (61.75%), pharmacists (12.85%), and other health professionals (19.70%), collectively accounting for 94.3% of all reports. Among reporting countries, China contributed the largest number of AE reports (n = 1,242, 51.30%), followed by the United States (n = 379, 15.65%). Regarding the reported indications, antiplatelet therapy was the most frequently cited (n = 1,430, 59.07%), followed by ischemic stroke (n = 220, 9.09%). A substantial proportion of patients experienced serious outcomes (n = 2,115, 87.36%), with death (n = 339, 14.00%), life-threatening events (n = 290, 11.98%), and hospitalizations (n = 242, 10.00%). These data are detailed in Supplementary Figure S1 and Supplementary Table S3. It is important to note that corresponding data on reporter identity, indication, and outcome are not available in the WHO-VigiAccess database.

### 3.2 Signal detection at the SOC level

AEs was categorized according to the System Organ Class (SOC) system. The number of PTs under each SOC was ranked in descending order. Tirofiban-related AEs were distributed across 24 SOCs in the FAERS dataset and 25 SOCs in the WHO-VigiAccess dataset, as detailed in Supplementary Tables S4, S5. Among these, 23 SOCs were shared between the two databases, as summarized in Table 2. Notably, three SOCs emerged as key contributors in both databases based on the disproportionality analysis. These included cardiac disorders (n = 862 in FAERS, n = 497 in WHO-VigiAccess, vascular disorders (n = 547 in FAERS, n = 648 in WHO-VigiAccess), and blood and lymphatic system disorders (n = 293 in FAERS, n = 1,569 in WHO-VigiAccess). In the FAERS database, cardiac disorders represented the most frequently reported and most strongly signaled SOC. In contrast, in the WHO-VigiAccess database, blood and lymphatic system disorders were the most prevalent and prominently signaled SOC.

**TABLE 2:** Overlap of tirofiban-related AEs by SOC across FAERS and WHO-VigiAccess databases.

System organ Class (SOC)	*FAERS*					*WHO-VigiAccess*				
	Case reports	ROR (95% CI)	PRR (χ2)	IC (IC025)	EBGM (EBGM05)	Case reports	ROR (95% CI)	PRR (χ2)	IC (IC025)	EBGM (EBGM05)
Cardiac disorders^a^	862	12.18 (11.28,13.16)	9.42 (6656.83)	3.23 (3.11)	9.41 (8.72)	497	3.26 (2.98,3.57)	3.09 (721.32)	1.63 (1.49)	3.09 (2.82)
Vascular disorders^a^	547	8.55 (7.80,9.36)	7.36 (3071.13)	2.88 (2.73)	7.36 (6.72)	648	4.86 (4.48,5.27)	4.49 (1794.99)	2.17 (2.04)	4.49 (4.14)
Nervous system disorders	414	1.45 (1.31,1.61)	1.40 (51.79)	0.49 (0.33)	1.40 (1.26)	493	0.69 (0.63,0.75)	0.71 (64.53)	−0.49 (-0.63)	0.71 (0.65)
General disorders and administration site conditions	386	0.59 (0.53,0.65)	0.63 (98.31)	−0.66 (-0.81)	0.63 (0.57)	722	0.47 (0.43,0.51)	0.53 (387.51)	−0.93 (-1.04)	0.53 (0.49)
Blood and lymphatic system disorders^a^	293	5.34 (4.74,6.02)	4.98 (947.09)	2.32 (2.12)	4.98 (4.42)	1569	13.37 (12.63,14.14)	10.50 (13783.0)	3.39 (3.30)	10.49 (9.92)
Respiratory, thoracic and mediastinal disorders	225	1.40 (1.22,1.60)	1.37 (23.72)	0.46 (0.25)	1.37 (1.20)	404	1.38 (1.25,1.53)	1.36 (39.71)	0.44 (0.29)	1.36 (1.23)
Investigations	183	0.85 (0.73,0.98)	0.86 (4.78)	−0.23 (-0.44)	0.86 (0.74)	442	1.04 (0.94,1.14)	1.03 (0.52)	0.05 (-0.09)	1.03 (0.94)
Injury, poisoning and procedural complications	153	0.40 (0.34,0.46)	0.42 (135.32)	−1.25 (-1.48)	0.42 (0.36)	264	0.59 (0.52,0.67)	0.61 (71.10)	−0.72 (-0.90)	0.61 (0.54)
Gastrointestinal disorders	144	0.46 (0.39,0.55)	0.49 (85.62)	−1.04 (-1.28)	0.49 (0.41)	838	1.28 (1.19,1.38)	1.25 (45.58)	0.32 (0.21)	1.25 (1.16)
Surgical and medical procedures	64	1.37 (1.07,1.75)	1.36 (6.13)	0.44 (0.07)	1.36 (1.06)	21	0.38 (0.25,0.58)	0.38 (21.10)	−1.39 (-1.96)	0.38 (0.25)
Renal and urinary disorders	52	0.78 (0.59,1.03)	0.78 (3.16)	−0.35 (-0.74)	0.78 (0.60)	262	2.47 (2.19,2.80)	2.42 (221.11)	1.27 (1.08)	2.42 (2.14)
Skin and subcutaneous tissue disorders	39	0.20 (0.14,0.27)	0.21 (124.92)	−2.27 (-2.70)	0.21 (0.15)	329	0.53 (0.47,0.59)	0.55 (132.61)	−0.86 (-1.02)	0.55 (0.49)
Infections and infestations	36	0.19 (0.14,0.26)	0.20 (124.68)	−2.35 (-2.79)	0.20 (0.14)	35	0.12 (0.09,0.17)	0.13 (214.48)	−2.95 (-3.40)	0.13 (0.09)
Product issues	22	0.38 (0.25,0.58)	0.39 (21.56)	−1.36 (-1.93)	0.39 (0.26)	8	0.13 (0.07,0.26)	0.13 (46.28)	−2.93 (-3.74)	0.13 (0.07)
Eye disorders	15	0.21 (0.13,0.35)	0.22 (43.62)	−2.21 (-2.86)	0.22 (0.13)	48	0.41 (0.31,0.54)	0.41 (41.31)	−1.28 (-1.68)	0.41 (0.31)
Musculoskeletal and connective tissue disorders	12	0.06 (0.04,0.11)	0.07 (165.74)	−3.91 (-4.60)	0.07 (0.04)	47	0.13 (0.09,0.17)	0.13 (283.58)	−2.92 (-3.31)	0.13 (0.10)
Immune system disorders	11	0.28 (0.16,0.51)	0.29 (19.81)	−1.81 (-2.55)	0.29 (0.16)	63	0.74 (0.58,0.95)	0.74 (5.80)	−0.43 (-0.79)	0.74 (0.58)
Hepatobiliary disorders	9	0.28 (0.15,0.54)	0.28 (16.69)	−1.83 (-2.64)	0.28 (0.15)	12	0.22 (0.13,0.39)	0.22 (32.88)	−2.17 (-2.88)	0.22 (0.13)
Psychiatric disorders	8	0.04 (0.02,0.08)	0.04 (191.24)	−4.62 (-5.42)	0.04 (0.02)	35	0.10 (0.07,0.15)	0.11 (267.69)	−3.20 (-3.64)	0.11 (0.08)
Metabolism and nutrition disorders	3	0.04 (0.01,0.12)	0.04 (71.27)	−4.66 (-5.70)	0.04 (0.01)	15	0.12 (0.07,0.20)	0.12 (94.67)	−3.01 (-3.65)	0.12 (0.07)
Reproductive system and breast disorders	3	0.10 (0.03,0.30)	0.10 (25.46)	−3.37 (-4.44)	0.10 (0.03)	7	0.10 (0.05,0.20)	0.10 (59.53)	−3.37 (-4.21)	0.10 (0.05)
Ear and labyrinth disorders	2	0.13 (0.03,0.53)	0.13 (11.43)	−2.92 (-4.09)	0.13 (0.03)	7	0.19 (0.09,0.40)	0.19 (23.98)	−2.38 (-3.25)	0.19 (0.09)
Neoplasms benign, malignant and unspecified (incl cysts and polyps)	2	0.02 (0.01,0.09)	0.02 (89.78)	−5.51 (-6.61)	0.02 (0.01)	2	0.02 (0.01,0.08)	0.02 (91.80)	−5.56 (-6.66)	0.02 (0.01)

^a^ SOCs, identified in both databases by all four disproportionality methods simultaneously.

ROR, reporting odds ratio; CI, confidence interval; PRR, proportional reporting ratio; IC, information component; IC025, the lower limit of 95% CI, of the IC; EBGM, empirical Bayesian geometric mean; EBGM05, the lower limit of 95% CI, of EBGM.

### 3.3 Distribution of AEs at the PT level

**FIGURE 3:**
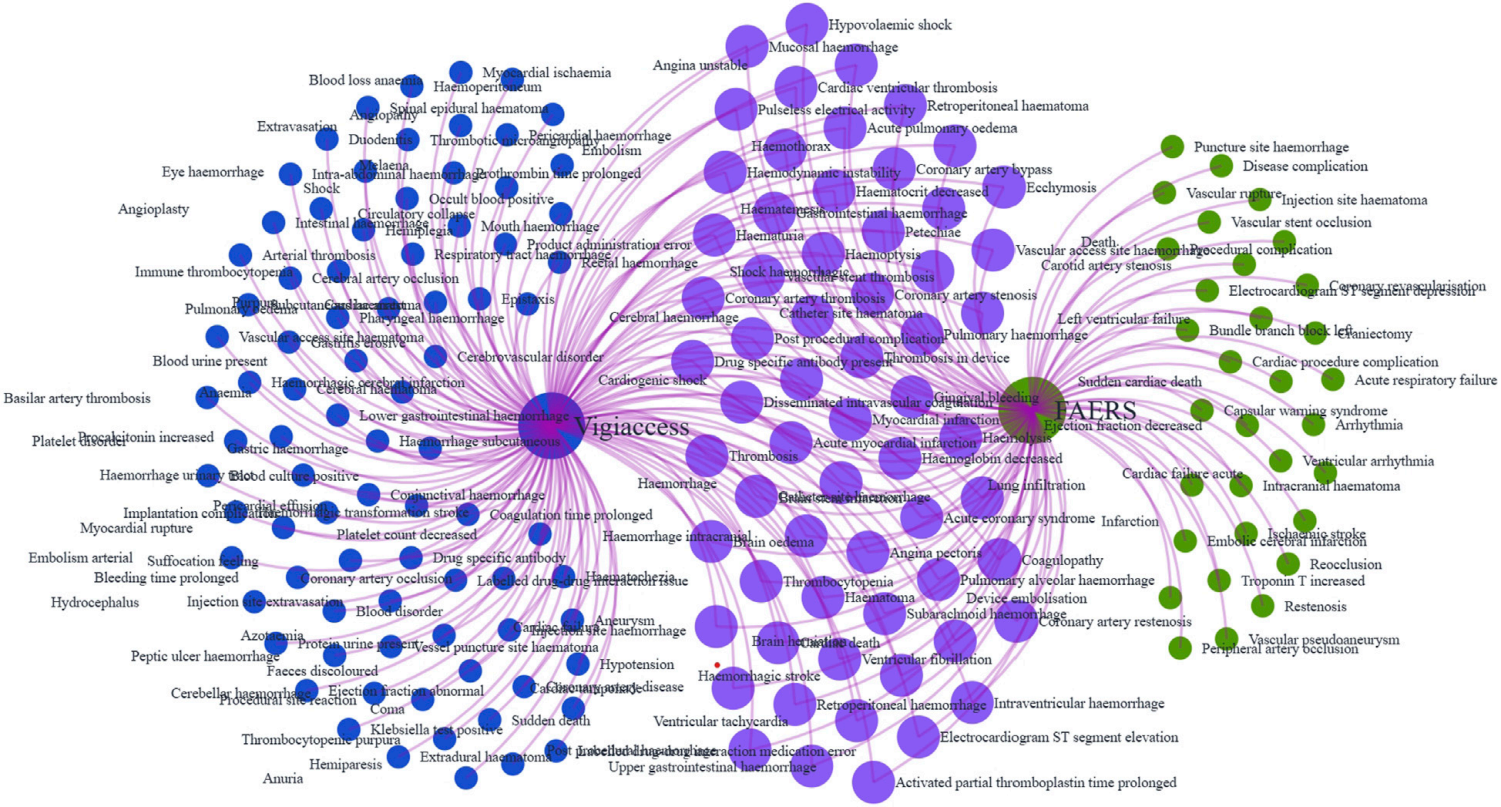
Venn diagram of tirofiban-related AE signals at the PT level identified in the FAERS and WHO-VigiAccess databases.

We further analyzed tirofiban-related AEs at the PT level. In the FAERS database, 95 PTs met all four disproportionality algorithm criteria simultaneously (Supplementary Table S6), while in the WHO-VigiAccess database, 147 PTs met the same criteria (Supplementary Table S7). A total of 63 PTs were identified in both datasets (Supplementary Table S8, in Figure 3). Among these overlapping PTs, commonly reported events included hemorrhage, thrombocytopenia, hematoma, petechiae and hemoptysis, align closely with the known adverse reactions listed in the tirofiban drug label. These 63 shared PTs were ranked by the frequency of reports in the FAERS database. The 30 most frequently reported PTs are presented in Table 3. The top five PTs by reporting frequency were: hemorrhage (n = 370 in FAERS, n = 223 in WHO-VigiAccess), thrombocytopenia (n = 248 in FAERS, n = 1,391 in WHO-VigiAccess), myocardial infarction (n = 207 in FAERS, n = 89 in WHO-VigiAccess), angina pectoris (n = 181 in FAERS, n = 26 in WHO-VigiAccess), and intracranial hemorrhage (n = 176 in FAERS, n = 77 in WHO-VigiAccess). In addition, the PTs were ranked based on signal strength using EBGM values in the FAERS database. The top 30 PTs with the highest EBGM signals are shown in Table 4. The three strongest signals were observed for vascular access site hemorrhage (6 cases, EBGM = 835.55 in FAERS, 4 cases, EBGM = 351.59 in WHO-VigiAccess),vascular stent thrombosis (55 cases, EBGM = 479.75 in FAERS, 10 cases, EBGM = 97.55 in WHO-VigiAccess), cardiac death (31 cases, EBGM = 437.46 in FAERS, 20 cases, EBGM = 283.22 in WHO-VigiAccess).

**TABLE 3:** Top 30 PTs in both databases ranked by FAERS reporting frequency (descending order).

Preferred terms	*FAERS*					*WHO-VigiAccess*				
	Case report	ROR (95% CI)	PRR (χ2)	IC (IC025)	EBGM (EBGM05)	Case report	ROR (95% CI)	PRR (χ2)	IC (IC025)	EBGM (EBGM05)
Haemorrhage	370	71.12 (63.84,79.23)	63.68 (22774.5)	5.99 (5.60)	63.43 (56.94)	223	28.48 (24.92,32.55)	27.58 (5709.44)	4.78 (4.43)	27.53 (24.09)
Thrombocytopenia	248	42.79 (37.60,48.70)	39.82 (9379.40)	5.31 (4.91)	39.72 (34.91)	1391	89.61 (84.47,95.06)	71.40 (96442.6)	6.15 (6.00)	71.11 (67.04)
Myocardial infarction^a^	207	20.95 (18.20,24.11)	19.76 (3693.82)	4.30 (3.97)	19.74 (17.15)	89	7.49 (6.08,9.24)	7.41 (493.92)	2.89 (2.48)	7.40 (6.01)
Angina pectoris^a^	181	113.77 (97.91,132.19)	107.91 (19051.5)	6.74 (5.86)	107.19 (92.25)	26	8.73 (5.94,12.83)	8.70 (177.13)	3.12 (2.20)	8.69 (5.91)
Haemorrhage intracranial	176	211.77 (181.81,246.66)	201.13 (34618.0)	7.63 (6.33)	198.63 (170.53)	77	56.01 (44.73,70.14)	55.39 (4099.89)	5.79 (4.70)	55.21 (44.09)
Cerebral haemorrhage	96	48.33 (39.44,59.21)	47.02 (4314.07)	5.55 (4.69)	46.89 (38.27)	105	29.47 (24.30,35.74)	29.03 (2838.33)	4.86 (4.24)	28.98 (23.90)
Cardiac failure^a^	80	17.95 (14.38,22.41)	17.56 (1249.56)	4.13 (3.54)	17.54 (14.05)	25	4.72 (3.19,6.99)	4.71 (73.02)	2.23 (1.47)	4.71 (3.18)
Vascular stent thrombosis^a^	55	502.58 (383.49,658.66)	494.67 (26278.9)	8.91 (5.26)	479.75 (366.07)	10	98.24 (52.74,182.99)	98.10 (955.71)	6.61 (2.45)	97.55 (52.37)
Cardiogenic shock^a^	38	49.84 (36.18,68.64)	49.30 (1793.13)	5.62 (3.99)	49.15 (35.69)	30	37.88 (26.45,54.24)	37.72 (1070.11)	5.23 (3.59)	37.64 (26.28)
Haemoglobin decreased	32	5.43 (3.84,7.70)	5.39 (114.64)	2.43 (1.75)	5.39 (3.81)	80	10.12 (8.12,12.62)	10.01 (649.45)	3.32 (2.85)	10.01 (8.03)
Cardiac death^a^	31	453.85 (317.10,649.56)	449.82 (13500.3)	8.77 (4.38)	437.46 (305.65)	20	288.73 (185.49,449.45)	287.88 (5624.77)	8.15 (3.66)	283.22 (181.94)
Gastrointestinal haemorrhage	29	5.89 (4.09,8.49)	5.85 (116.69)	2.55 (1.80)	5.85 (4.06)	138	16.28 (13.76,19.28)	15.97 (1937.50)	4.00 (3.60)	15.96 (13.48)
Acute myocardial infarction^a^	25	14.43 (9.73,21.38)	14.33 (309.89)	3.84 (2.67)	14.32 (9.66)	17	8.68 (5.39,13.97)	8.66 (115.11)	3.11 (1.92)	8.65 (5.38)
Coronary artery stenosis^a^	24	80.28 (53.68,120.06)	79.74 (1856.83)	6.31 (3.68)	79.34 (53.05)	10	37.80 (20.31,70.33)	37.74 (356.92)	5.24 (2.25)	37.66 (20.24)
Haematoma	24	15.94 (10.66,23.81)	15.83 (333.32)	3.98 (2.73)	15.82 (10.59)	147	40.25 (34.18,47.41)	39.40 (5491.92)	5.30 (4.72)	39.31 (33.38)
Haematuria	23	11.62 (7.71,17.51)	11.55 (221.57)	3.53 (2.41)	11.54 (7.66)	190	44.02 (38.10,50.86)	42.81 (7745.08)	5.42 (4.92)	42.71 (36.97)
Haemoptysis	20	12.55 (8.09,19.49)	12.49 (211.30)	3.64 (2.38)	12.48 (8.04)	88	36.81 (29.82,45.43)	36.34 (3019.10)	5.18 (4.39)	36.27 (29.38)
Pulmonary alveolar haemorrhage	19	62.68 (39.90,98.47)	62.34 (1142.43)	5.96 (3.29)	62.10 (39.53)	20	62.36 (40.17,96.79)	62.17 (1199.58)	5.95 (3.36)	61.96 (39.91)
Thrombosis^a^	18	3.91 (2.46,6.21)	3.90 (38.78)	1.96 (1.09)	3.89 (2.45)	25	4.23 (2.86,6.26)	4.22 (61.39)	2.08 (1.34)	4.22 (2.85)
Thrombosis in device	18	90.34 (56.78,143.75)	89.88 (1573.28)	6.48 (3.32)	89.38 (56.17)	4	20.49 (7.68,54.65)	20.48 (74.03)	4.35 (0.77)	20.46 (7.67)
Embolism^a^	17	35.39 (21.97,57.03)	35.23 (564.15)	5.14 (2.92)	35.15 (21.81)	13	17.43 (10.12,30.05)	17.40 (200.80)	4.12 (2.23)	17.39 (10.09)
Catheter site haemorrhage	15	110.15 (66.22,183.23)	109.68 (1604.32)	6.77 (3.09)	108.93 (65.49)	15	92.80 (55.84,154.23)	92.60 (1352.00)	6.53 (3.06)	92.12 (55.43)
Petechiae	14	23.96 (14.17,40.51)	23.86 (306.27)	4.57 (2.49)	23.83 (14.09)	37	17.75 (12.85,24.52)	17.66 (581.07)	4.14 (3.15)	17.64 (12.77)
Ventricular fibrillation^a^	14	22.04 (13.04,37.27)	21.96 (279.72)	4.45 (2.45)	21.93 (12.97)	17	17.56 (10.91,28.28)	17.52 (264.60)	4.13 (2.51)	17.50 (10.87)
Post procedural complication	14	12.61 (7.46,21.31)	12.56 (148.90)	3.65 (2.08)	12.55 (7.42)	11	11.04 (6.11,19.95)	11.02 (100.19)	3.46 (1.75)	11.02 (6.10)
Gingival bleeding	13	16.78 (9.73,28.94)	16.72 (192.02)	4.06 (2.20)	16.71 (9.69)	157	105.86 (90.32,124.07)	103.43 (15834.9)	6.68 (5.73)	102.82 (87.73)
Retroperitoneal haemorrhage	13	88.48 (51.25,152.76)	88.15 (1114.00)	6.45 (2.83)	87.67 (50.78)	40	129.26 (94.62,176.58)	128.50 (5023.60)	7.00 (4.51)	127.57 (93.38)
Haematocrit decreased	11	9.37 (5.18,16.93)	9.34 (81.91)	3.22 (1.63)	9.34 (5.16)	32	19.31 (13.64,27.33)	19.22 (552.24)	4.26 (3.13)	19.20 (13.56)
Drug specific antibody present^a^	10	27.71 (14.89,51.58)	27.64 (256.31)	4.79 (2.14)	27.59 (14.82)	8	22.67 (11.33,45.36)	22.64 (165.28)	4.50 (1.77)	22.61 (11.30)
Pulmonary haemorrhage	10	21.86 (11.74,40.67)	21.80 (198.18)	4.44 (2.04)	21.77 (11.70)	50	81.10 (61.36,107.18)	80.51 (3908.31)	6.32 (4.57)	80.14 (60.64)

^a^ Not mentioned in the drug label.

**TABLE 4:** Top 30 PTs with the strongest AE signals in both databases ranked by ROR in the FAERS database (Descending Order).

Preferred terms	*FAERS*					*WHO-VigiAccess*				
	Case report	ROR (95% CI)	PRR (χ2)	IC (IC025)	EBGM (EBGM05)	Case report	ROR (95% CI)	PRR (χ2)	IC (IC025)	EBGM (EBGM05)
Vascular access site haemorrhage	6	883.43 (388.02,2011.38)	881.91 (5001.61)	9.71 (1.67)	835.55 (366.99)	4	359.03 (133.36,966.56)	358.82 (133.36,965.43)	8.46 (1.00)	351.59 (130.60)
Vascular stent thrombosis^a^	55	502.58 (383.49,658.66)	494.67 (26278.9)	8.91 (5.26)	479.75 (366.07)	10	98.24 (52.74,182.99)	98.10 (52.71,182.56)	6.61 (2.45)	97.55 (52.37)
Cardiac death^a^	31	453.85 (317.10,649.56)	449.82 (13500.3)	8.77 (4.38)	437.46 (305.65)	20	288.73 (185.49,449.45)	287.88 (185.18,447.55)	8.15 (3.66)	283.22 (181.94)
Catheter site haematoma	5	308.08 (127.07,746.94)	307.64 (1499.19)	8.24 (1.37)	301.81 (124.49)	5	237.27 (98.14,573.64)	237.09 (98.13,572.85)	7.87 (1.37)	233.92 (96.75)
Haemorrhage intracranial	176	211.77 (181.81,246.66)	201.13 (34618.0)	7.63 (6.33)	198.63 (170.53)	77	56.01 (44.73,70.14)	55.39 (44.34,69.18)	5.79 (4.70)	55.21 (44.09)
Catheter site haemorrhage	15	110.15 (66.22,183.23)	109.68 (1604.32)	6.77 (3.09)	108.93 (65.49)	15	92.80 (55.84,154.23)	92.60 (55.78,153.72)	6.53 (3.06)	92.12 (55.43)
Angina pectoris^a^	181	113.77 (97.91,132.19)	107.91 (19051.5)	6.74 (5.86)	107.19 (92.25)	26	8.73 (5.94,12.83)	8.70 (5.93,12.77)	3.12 (2.20)	8.69 (5.91)
Coronary artery restenosis^a^	4	106.84 (39.94,285.77)	106.72 (416.12)	6.73 (0.97)	106.01 (39.63)	3	79.14 (25.45,246.07)	79.10 (25.45,245.84)	6.30 (0.50)	78.75 (25.33)
Thrombosis in device^a^	18	90.34 (56.78,143.75)	89.88 (1573.28)	6.48 (3.32)	89.38 (56.17)	4	20.49 (7.68,54.65)	20.48 (7.68,54.58)	4.35 (0.77)	20.46 (7.67)
Retroperitoneal haemorrhage	13	88.48 (51.25,152.76)	88.15 (1114.00)	6.45 (2.83)	87.67 (50.78)	40	129.26 (94.62,176.58)	128.50 (94.24,175.23)	7.00 (4.51)	127.57 (93.38)
Coronary artery stenosis^a^	24	80.28 (53.68,120.06)	79.74 (1856.83)	6.31 (3.68)	79.34 (53.05)	10	37.80 (20.31,70.33)	37.74 (20.30,70.16)	5.24 (2.25)	37.66 (20.24)
Coronary artery thrombosis^a^	8	66.54 (33.20,133.35)	66.39 (513.08)	6.05 (2.04)	66.11 (32.99)	10	57.61 (30.95,107.24)	57.53 (30.94,106.98)	5.84 (2.36)	57.34 (30.81)
Haemorrhage	370	71.12 (63.84,79.23)	63.68 (22774.5)	5.99 (5.60)	63.43 (56.94)	223	28.48 (24.92,32.55)	27.58 (24.23,31.38)	4.78 (4.43)	27.53 (24.09)
Pulmonary alveolar haemorrhage	19	62.68 (39.90,98.47)	62.34 (1142.43)	5.96 (3.29)	62.10 (39.53)	20	62.36 (40.17,96.79)	62.17 (40.11,96.38)	5.95 (3.36)	61.96 (39.91)
Cardiogenic shock^a^	38	49.84 (36.18,68.64)	49.30 (1793.13)	5.62 (3.99)	49.15 (35.69)	30	37.88 (26.45,54.24)	37.72 (26.38,53.92)	5.23 (3.59)	37.64 (26.28)
Brain stem infarction^a^	3	47.95 (15.43,149.00)	47.91 (137.38)	5.58 (0.47)	47.77 (15.37)	3	32.79 (10.56,101.80)	32.77 (10.56,101.70)	5.03 (0.43)	32.71 (10.54)
Cerebral haemorrhage	96	48.33 (39.44,59.21)	47.02 (4314.07)	5.55 (4.69)	46.89 (38.27)	105	29.47 (24.30,35.74)	29.03 (24.01,35.10)	4.86 (4.24)	28.98 (23.90)
Mucosal haemorrhage	3	45.44 (14.62,141.18)	45.40 (129.89)	5.50 (0.46)	45.27 (14.57)	7	81.62 (38.83,171.58)	81.54 (38.82,171.27)	6.34 (1.86)	81.16 (38.61)
Thrombocytopenia	248	42.79 (37.60,48.70)	39.82 (9379.40)	5.31 (4.91)	39.72 (34.91)	1391	89.61 (84.47,95.06)	71.40 (68.13,74.84)	6.15 (6.00)	71.11 (67.04)
Cardiac ventricular thrombosis^a^	9	36.09 (18.75,69.47)	36.00 (305.55)	5.17 (2.08)	35.92 (18.66)	8	27.93 (13.95,55.91)	27.90 (13.95,55.80)	4.80 (1.84)	27.86 (13.92)
Embolism^a^	17	35.39 (21.97,57.03)	35.23 (564.15)	5.14 (2.92)	35.15 (21.81)	13	17.43 (10.12,30.05)	17.40 (10.11,29.96)	4.12 (2.23)	17.39 (10.09)
Pulseless electrical activity^a^	8	28.69 (14.32,57.44)	28.62 (212.89)	4.84 (1.85)	28.57 (14.27)	5	16.21 (6.74,38.98)	16.20 (6.74,38.93)	4.02 (1.02)	16.19 (6.73)
Drug specific antibody present^a^	10	27.71 (14.89,51.58)	27.64 (256.31)	4.79 (2.14)	27.59 (14.82)	8	22.67 (11.33,45.36)	22.64 (11.32,45.28)	4.50 (1.77)	22.61 (11.30)
Brain herniation^a^	4	23.92 (8.96,63.80)	23.89 (87.60)	4.58 (0.81)	23.85 (8.94)	6	34.39 (15.43,76.63)	34.36 (15.43,76.51)	5.10 (1.48)	34.29 (15.39)
Petechiae	14	23.96 (14.17,40.51)	23.86 (306.27)	4.57 (2.49)	23.83 (14.09)	37	17.75 (12.85,24.52)	17.66 (12.80,24.36)	4.14 (3.15)	17.64 (12.77)
Retroperitoneal haematoma	3	23.65 (7.62,73.44)	23.63 (64.94)	4.56 (0.38)	23.60 (7.60)	3	15.23 (4.91,47.26)	15.23 (4.91,47.22)	3.93 (0.30)	15.21 (4.90)
Cardiac tamponade^a^	6	22.09 (9.91,49.24)	22.06 (120.46)	4.46 (1.37)	22.03 (9.88)	17	60.62 (37.63,97.66)	60.48 (37.59,97.30)	5.91 (3.13)	60.27 (37.41)
Ventricular fibrillation^a^	14	22.04 (13.04,37.27)	21.96 (279.72)	4.45 (2.45)	21.93 (12.97)	17	17.56 (10.91,28.28)	17.52 (10.90,28.17)	4.13 (2.51)	17.50 (10.87)
Pulmonary haemorrhage	10	21.86 (11.74,40.67)	21.80 (198.18)	4.44 (2.04)	21.77 (11.70)	50	81.10 (61.36,107.18)	80.51 (61.04,106.18)	6.32 (4.57)	80.14 (60.64)
Electrocardiogram ST segment elevation^a^	4	20.68 (7.75,55.16)	20.66 (74.73)	4.37 (0.77)	20.63 (7.73)	4	13.92 (5.22,37.13)	13.92 (5.22,37.08)	3.80 (0.67)	13.91 (5.22)

^a^ Not mentioned in the drug label.

In addition, besides the common AEs listed in the tirofiban drug insert, we also identified 21 suspected AEs not mentioned in the drug label as shown in Supplementary Table S8, including myocardial infarction (207 cases, EBGM = 19.74 in FAERS,89 cases, EBGM = 7.4 in WHO-VigiAccess), angina pectoris (181 cases, EBGM = 107.19 in FAERS,26 cases, EBGM = 8.69 in WHO-VigiAccess), cardiac failure (80 cases, EBGM = 17.54 in FAERS, 25 cases, EBGM = 4.71 in WHO-VigiAccess), vascular stent thrombosis (55 cases, EBGM = 479.75 in FAERS, 10 cases, EBGM = 97.55 in WHO-VigiAccess), cardiogenic shock (38 cases, EBGM = 49.15 in FAERS, 30 cases, EBGM = 37.64 in WHO-VigiAccess), cardiac death (31 cases, EBGM = 437.46 in FAERS, 20cases, EBGM = 283.22 in WHO-VigiAccess), acute myocardial infarction (25 cases, EBGM = 14.32 in FAERS, 17 cases, EBGM = 8.65 in WHO-VigiAccess), coronary artery stenosis (24 cases, EBGM = 79.34 in FAERS, 10 cases, EBGM = 37.66 in WHO-VigiAccess), thrombosis (18 cases, EBGM = 3.89 in FAERS, 25 cases, EBGM = 4.22 in WHO-VigiAccess), embolism (17 cases, EBGM = 35.15 in FAERS, 13 cases, EBGM = 17.39 in WHO-VigiAccess), ventricular fibrillation (14 cases, EBGM = 21.93 in FAERS, 17 cases, EBGM = 17.50 in WHO-VigiAccess), drug specific antibody present (10 cases, EBGM = 27.59 in FAERS, 8 cases, EBGM = 22.61 in WHO-VigiAccess), coronary artery restenosis (4 cases, EBGM = 106.01 in FAERS, 3 cases, EBGM = 78.75 in WHO-VigiAccess),thrombosis in device (18 cases, EBGM = 89.38 in FAERS, 4 cases, EBGM = 20.46 in WHO-VigiAccess),coronary artery thrombosis (8 cases, EBGM = 66.11 in FAERS, 10 cases, EBGM = 57.34 in WHO-VigiAccess), brain stem infarction (3 cases, EBGM = 47.77 in FAERS, 3 cases, EBGM = 32.71 in WHO-VigiAccess), cardiac ventricular thrombosis (9 cases, EBGM = 35.92 in FAERS, 8 cases, EBGM = 27.86 in WHO-VigiAccess), pulseless electrical activity (8 cases, EBGM = 28.57 in FAERS, 5 cases, EBGM = 16.19 in WHO-VigiAccess), brain herniation (4 cases, EBGM = 23.85 in FAERS, 6 cases, EBGM = 34.29 in WHO-VigiAccess), cardiac tamponade (6 cases, EBGM = 22.03 in FAERS, 17 cases, EBGM = 60.27 in WHO-VigiAccess) and electrocardiogram ST segment elevation (4 cases, EBGM = 20.63 in FAERS, 4 cases, EBGM = 13.91 in WHO-VigiAccess).

### 3.4 Time to onset of tirofiban-related AEs

**FIGURE 4:**
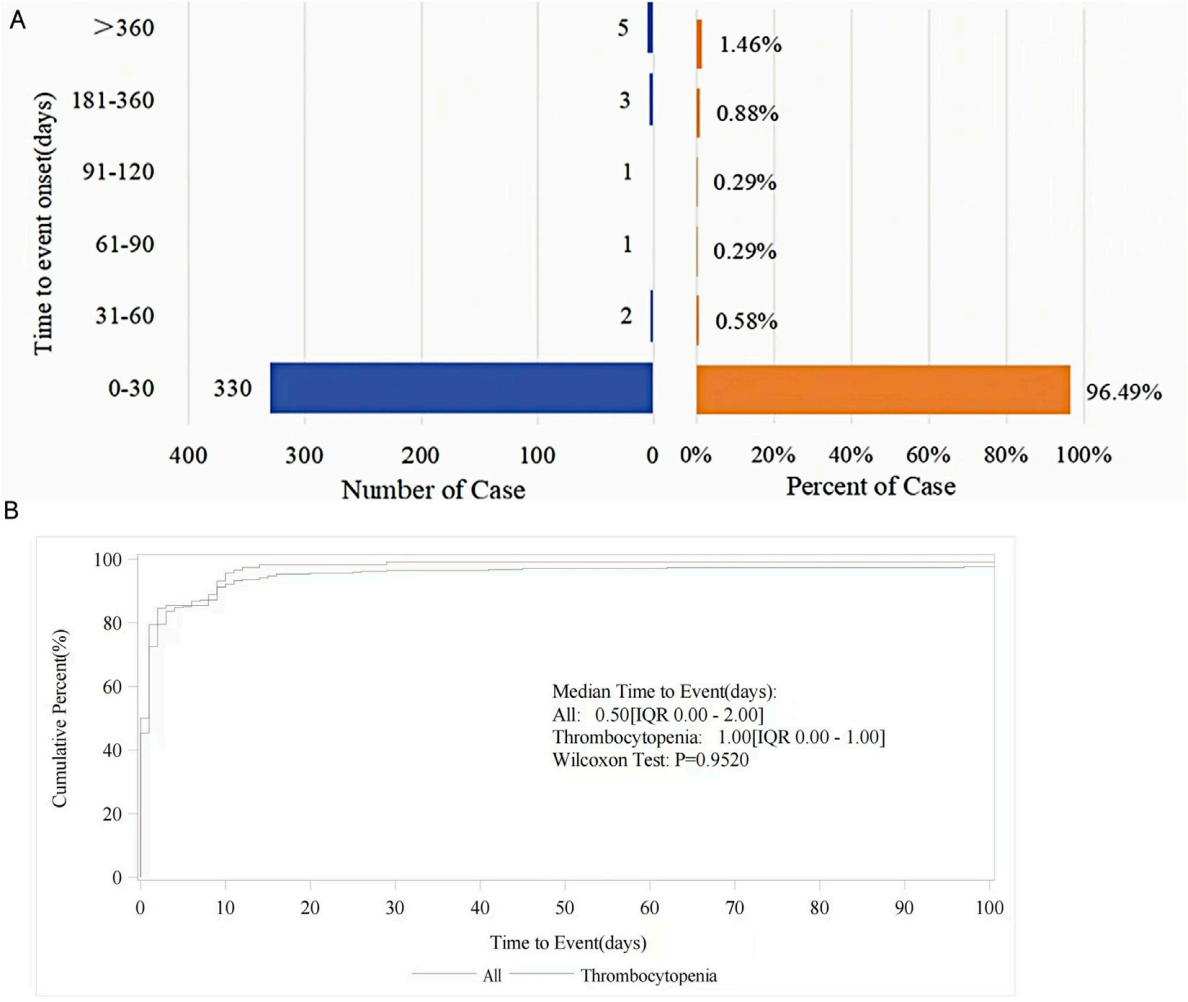
**(A)** Distribution of AE onset time in reported cases. **(B)** Cumulative incidence curves for tirofiban-related AEs.

In the FAERS database, the onset time of tirofiban-related AEs was recorded after excluding missing data or outliers. A total of 342 AEs reported the time of onset, with the majority occurring within 0–30 days (330 cases, 96.49%) (Figure 4A). The median onset time was 0.5 days (12 h) (IQR 0–2 days). For the top three most frequent PTs, the median onset time for hemorrhage and intracranial hemorrhage was very brief, at 0.00 days (IQR, 0.00,0.00), while the median onset time for thrombocytopenia was 1.00 days (IQR, 0.00,1.00) (Table 5; Figure 4B). Data on onset time are not available in the WHO-VigiAccess database. Figure 4B shows the cumulative incidence curves for all AEs, including thrombocytopenia.

**TABLE 5:** Weibull distribution and failure types for the top 3 most frequent PTs and total onset time of AEs.

PT	Available cases (n)	Time to event (day,median (Q1,Q3))	Weibull distribution		Failure type
			α(95%)	β(95%)	
Haemorrhage	16	0.00 (0.00,0.00)	13.599 (2.845,64.996)	0.935 (0.295,2.970	Random failure
Thrombocytopenia	117	1.00 (0.00,1.00)	4.193 (2.707,6.495)	0.597 (0.513,0.694)	Early failure
Haemorrhage intracranial^a^	5	0.00 (0.00,0.00)	-	-	-
Overall	342	0.50 (0.00,2.00)	7.772 (5.335,11.324)	0.424 (0.388,0.464)	Early failure

^a^ Relatively small number of cases, not analyzed by Weibull distribution. IQR, interquartile range.

Furthermore, the Weibull distribution test for TTO revealed that the lower bounds of the 95% CI for the shape parameter (β) were less than 1 for both thrombocytopenia and total cases, suggesting indicating an early failure type. This indicates a gradual decline in the probability of AE over time. In contrast, bleeding events were identified as having a random failure type.

### 3.5 Sensitivity analysis

In the stratified analysis based on reporter type within the FAERS database (presented in Supplementary Table S9), we found that the AE signals identified from healthcare professional reports were largely consistent with those derived from all reporters. The five most frequently reported PTs remained hemorrhage (367 cases, EBGM = 69.41), thrombocytopenia (220 cases, EBGM = 22.57), myocardial infarction (204 cases, EBGM = 26.21), angina pectoris (179 cases, EBGM = 105.58), and intracranial hemorrhage (175 cases, EBGM = 134.22).

In the gender-difference analysis (Supplementary Table S10), we observed that thrombocytopenia showed a significantly stronger signal in male patients than female patients (ROR 0.47, 95% CI 0.33–0.69, P < 0.001). In contrast, female patients appeared to have a higher likelihood of experiencing angina pectoris (ROR 2.72, 95% CI 1.43–5.15, P = 0.0015), chest pain (ROR 4.46, 95% CI 1.20–16.52, P = 0.0316) and coronary artery stenosis (ROR 3.64, 95% CI 1.50–8.82, P = 0.0023). However, for the PTs about bleeding, including haemorrhage, cerebral haemorrhage, pulmonary alveolar haemorrhage, gastrointestinal haemorrhage, and haemoglobin decreased, we found no statistically significant differences between different gender (P > 0.05).

The results of the age-stratified analysis are shown in Table 6. For most AEs, such as thrombocytopenia, myocardial infarction, and death, we did not observe statistically significant differences between patients aged <65 years and those aged ≥65 years (P > 0.05). However, two notable heterogeneous signals were identified: thrombosis in device showed a significantly stronger signal in younger patients (ROR = 3.53, 95% CI 1.08–11.52, *P* = 0.0259), whereas hemoglobin decreased was reported significantly more frequently in older patients (ROR = 0.37, 95% CI 0.15–0.90, *P* = 0.0221).

**TABLE 6:** Age-stratified analysis of specific PTs associated with tirofiban in the FAERS database.

Preferred term (PT)	<65 years (N)	≥65 years (N)	ROR (95% CI)	χ^2^	P
Thrombocytopenia	47	86	0.83 (0.57,1.21)	0.9211	0.3372
Myocardial infarction	18	28	1.00 (0.55,1.82)	0.0001	0.9916
Haemoglobin decreased	6	25	0.37 (0.15,0.90)	5.2400	**0.0221**
Cardiogenic shock	11	17	1.00 (0.47,2.16)	0.0001	0.9929
Death	8	17	0.73 (0.31,1.69)	0.5543	0.4566
Coronary artery stenosis	10	13	1.20 (0.52,2.75)	0.1792	0.6721
Acute myocardial infarction	9	14	1.00 (0.43,2.32)	0.0001	0.9941
Haemorrhage	6	17	0.54 (0.21,1.38)	1.6880	0.1939
Cerebral haemorrhage	4	14	0.44 (0.14,1.34)	2.2062	0.1375
Pulmonary alveolar haemorrhage	5	11	0.70 (0.24,2.03)	0.4296	0.5122
Haematoma	4	12	0.51 (0.16,1.60)	1.3700	0.2418
Ventricular fibrillation	6	8	1.16 (0.40,3.38)	0.0792	0.7784
Platelet count decreased	4	10	0.62 (0.19,1.98)	0.6708	0.4128
Hypotension	8	6	2.08 (0.72,6.04)	1.9091	0.1671
Cardiac failure	4	9	0.69 (0.21,2.24)	0.3917	0.5314
Post procedural complication	5	8	0.97 (0.32,2.98)	0.0030	0.9560
Thrombosis in device	9	4	**3.53 (1.08,11.52)**	4.9632	**0.0259**
Haemoptysis	3	10	0.46 (0.13,1.69)	1.4319	0.2315
Chest pain	3	9	0.51 (0.14,1.91)	1.0246	0.4744^a^
Petechiae	2	10	0.31 (0.07,1.14)	2.5794	0.1905^a^
Cardiac arrest	5	6	1.29 (0.39,4.26)	0.1817	0.9075^a^
Gastrointestinal haemorrhage	3	8	0.58 (0.15,2.19)	0.6622	0.6144^a^
Anaemia	3	7	0.66 (0.17,2.57)	0.3580	0.7846^a^
Vascular stent thrombosis	6	4	2.34 (0.66,8.33)	1.8274	0.3045^a^
Pyrexia	3	7	0.66 (0.17,2.57)	0.3580	0.7846^a^
Drug ineffective	3	7	0.66 (0.17,2.57)	0.3580	0.7846^a^
Drug specific antibody present	3	7	0.66 (0.17,2.57)	0.3580	0.7846^a^
Ejection fraction decreased	3	7	0.66 (0.17,2.57)	0.3580	0.7846^a^
Haematocrit decreased	2	8	0.39 (0.08,1.82)	1.5585	0.3558^a^
Haemorrhage intracranial	4	3	2.08 (0.46,9.31)	0.9498	0.5575^a^

^a^ Yates’ continuity-corrected chi-square test. Bold values indicate statistical significance (P < 0.05) unless otherwise specified.

Abbreviations: FAERS, the U.S., Food and Drug Administration’s Adverse Event Reporting System; PT, preferred term; ROR, reporting odds ratio; CI, confidence interval. χ^2^:chi-square.

## 4 Discussion

In our study, AE signals associated with tirofiban were identified from two databases and were found to involve 23 SOCs and 63 PTs, primarily concentrated in the SOCs of cardiac disorders, vascular disorders, and blood and lymphatic system disorders. The top 3 most frequently reported AEs were hemorrhage, thrombocytopenia, and intracranial hemorrhage, which are consistent with the AEs listed in the drug prescribing information, thereby supporting the reliability of our findings. In addition, this study identified 21 AEs that were not mentioned in the official drug label, including myocardial infarction, angina pectoris, cardiac failure, vascular stent thrombosis, cardiogenic shock, cardiac death, acute myocardial infarction, coronary artery stenosis, thrombosis, embolism, ventricular fibrillation, drug specific antibody present, coronary artery restenosis, thrombosis in device, coronary artery thrombosis, brain stem infarction, cardiac ventricular thrombosis, pulseless electrical activity, brain herniation, cardiac tamponade and electrocardiogram ST segment elevation.

### 4.1 Baseline information description

This study systematically analyzed the baseline characteristics of tirofiban-associated AEs reported in the FAERS and WHO-VigiAccess databases, revealing notable differences between the two data sources. Both databases showed a higher proportion of AEs reported in elderly and male patients. These findings align with existing literature, which reports a higher prevalence of cardiovascular diseases, particularly among older patients, with a greater incidence in males compared to females (Roth et al., 2021; Mensah et al., 2023). Our data analysis supports these epidemiological trends. As shown in Figure 2, a marked increase in the number of tirofiban-related adverse event reports was observed in 2019. This phenomenon may be related to the increased use of the drug (e.g., its exploratory application in ischemic stroke, such as in the RESCUE BT randomized clinical trial), which may have heightened clinical attention and temporarily increased reporting rates. It may also reflect an improvement in adverse event reporting awareness during this period.

Among the AE reports in the FAERS database, a significant proportion (94.3%) were submitted by healthcare professionals, including physicians, pharmacists, and other health professionals. This suggests that the FAERS database is a reliable source of AE reporting. In terms of geographic distribution, China accounted for the highest number of reports. This may be attributed to several factors, including a larger population of drug users, greater overall population size, racial disparities, a higher willingness to report, and the broader indications for tirofiban, all contributing to its more widespread use in this region. Regarding reported indications, the majority of AEs were associated with antiplatelet therapy, which aligns with its primary indication. Ischemic stroke followed as the second most reported indication. This finding is consistent with recent studies demonstrating the significant efficacy and safety of tirofiban in the treatment of acute ischemic stroke (Sun et al., 2021; Zi et al., 2023). These findings suggest that, while tirofiban is effective in acute clinical settings, its use necessitates careful patient selection, monitoring, and management to mitigate risks.

### 4.2 SOC for which both databases satisfy the thresholds

In both databases, we identified 23 organ systems commonly affected by tirofiban-related AEs. Among these, significant SOCs that met all four disproportionality analysis criteria included cardiac disorders, vascular and lymphovascular disorders, and hematologic and lymphatic disorders. Notably, cardiac disorders were the most frequently reported and highest signaling SOC in the FAERS database, while blood and lymphatic system disorders were the most common and highest signaling SOC in the WHO-VigiAccess database. These findings highlight the significant risk of cardiovascular and hematologic complications associated with tirofiban, consistent with the drug’s known mechanism as an antiplatelet agent.

#### 4.2.1 AEs related to cardiac disorders

Based on the drug label for tirofiban, hemopericardium is a commonly reported AE associated with cardiac disorders. However, our study identified that the most frequently reported PTs related to cardiac disorders among the top 30 AEs were myocardial infarction, angina pectoris, cardiac failure, cardiogenic shock, acute myocardial infarction, coronary artery stenosis, and ventricular fibrillation. Notably, myocardial infarction, angina pectoris, acute myocardial infarction, and coronary artery stenosis are recognized indications for tirofiban (De et al., 2009; Valgimigli et al., 2010; Deng et al., 2021; Zhao et al., 1999; PRISM-PLUS Study Investigators, 1998), which may introduce bias at the SOC level in the analysis of cardiac disorders. Cardiac failure may also represent a comorbidity of the patients’ underlying conditions rather than a direct effect of tirofiban. For instance, myocardial infarction is primarily caused by rupture of atherosclerotic plaques in the coronary arteries, leading to platelet aggregation and thrombosis. The subsequent occlusion of coronary vessels results in myocardial ischemia, which can cause interstitial fibrosis, progressive ventricular dilation, and ultimately heart failure, thereby compromising patients’ quality of life and safety (Zhuo et al., 2022). Similarly, cardiogenic shock and ventricular fibrillation may be complications secondary to the underlying disease or its treatment. There have been case reports of tirofiban being used in patients with acute myocardial infarction complicated by cardiogenic shock (Knezević et al., 2008; Canji et al., 2010). Moreover, a randomized, prospective, open-label trial indicated that tirofiban, when used in addition to unfractionated heparin (UFH), could prevent platelet depletion and preserve platelet function in patients with cardiogenic shock and acute kidney injury requiring continuous renal replacement therapy (CRRT) (Link et al., 2008). Based on the data from both databases and disproportionality analysis, cardiac-related adverse events such as cardiac failure, cardiogenic shock, and ventricular fibrillation showed strong disproportionality signals. However, these findings should be interpreted with caution, as the statistical association observed in spontaneous reporting systems does not establish a causal relationship. The presence of underlying cardiovascular conditions and concomitant therapies may contribute to these signals. Therefore, these events should be considered potential safety signals rather than confirmed adverse drug reactions, and further clinical and mechanistic studies are warranted to clarify their clinical relevance.

#### 4.2.2 AEs related to vascular disorders

Tirofiban treatment may lead to a range of vascular disorders, including bleeding and hematoma at various anatomical sites, with a higher incidence of hemorrhagic events when used in combination with heparin. Typically, bleeding events associated with tirofiban are mild, whereas severe cases, such as intracranial hemorrhage and retroperitoneal bleeding, are exceedingly rare. In addition to detecting signals of bleeding and intracranial hemorrhage, our study also identified thrombotic and embolic events, which are not mentioned in the drug label. From the pharmacological perspective, a clinical study reported a 10.5% incidence of minor bleeding with tirofiban, whereas the incidence of major bleeding, as defined by the thrombolysis in myocardial infarction (TIMI) criteria, was 1.4% (Huynh et al., 2003). For intracranial hemorrhage, in an analysis evaluating the safety outcomes of tirofiban in acute ischemic stroke treatment, the incidence of symptomatic intracranial hemorrhage (sICH) within 7 days was 0.6%, and any bleeding occurred in 4.0% of patients (Han et al., 2022). Moreover, intracranial hemorrhage has been reported in multiple studies following the use of tirofiban (Ihn et al., 2011; Liu et al., 2022; Zhang et al., 2020; De Almeida Monteiro et al., 2025), with fatal intracranial hemorrhage in some studies (relative risk = 3.59) (Liu et al., 2022). A recent phase III, multicenter, double-blind, randomized, placebo-controlled trial showed that the incidence of intracranial hemorrhage associated with tirofiban was relatively low (1.7%), although still higher than that observed in the placebo group (Chunrong et al., 2025). There are case reports of tirofiban-induced alveolar hemorrhage (Sebastian et al., 2024). The RESCUE BT study reported that older patients, had more severe neurological deficits, or had cardioembolic stroke were at a higher risk of developing sICH following intravenous tirofiban administration (Sang et al., 2023b). In addition, haemorrhage associated with tirofiban often occurs at arterial access sites used in cardiac catheterization procedures. It is recommended to minimize the use of invasive or potentially traumatic procedures, such as arterial and venous puncture, intramuscular injection, and nasotracheal intubation. Apart from drug-related factors, bleeding is also influenced by patient specific factors, such as advanced age, sex (particularly small-statured females), and comorbidities, including renal impairment, a history of prior bleeding, or malignancy (Chang et al., 2024; Sameer et al., 2009). Consequently, bleeding risk assessment should be performed before tirofiban administration, and patients should be closely monitored throughout treatment.

Regarding other vascular AEs, such as thrombosis and embolism, current evidence suggests that pre-procedural intravenous administration of tirofiban can reduce the risk of acute in-stent thrombosis during stenting procedures in patients with symptomatic high-grade intracranial atherosclerotic stenosis (Zhang et al., 2024). Some studies have reported that in intra-arterial strategies using tirofiban for the treatment of acute ischemic stroke, there is a risk of reocclusion following intra-arterial thrombolysis (Kim et al., 2012). A study showed that in-stent thrombosis occurred at an annual rate of 3% within 0.6 days–3 years post-implantation (Daemen et al., 2007). According to the FDA, various factors contribute to the formation of in-stent thrombosis, including the implantation of drug-eluting stents in patients with complex lesions (e.g., bifurcations, lesions requiring overlapping stents, or lesions associated with acute myocardial infarction), as well as conditions such as renal insufficiency or diabetes, which can lead to a higher incidence of stent thrombosis compared to implantation for approved indications. The FDA also notes a small increase in risk (ranging from <1% to approximately 5%) due to premature discontinuation of antiplatelet therapy, which remains an independent risk factor for thrombosis. Notably, thrombosis can occur even years after stent implantation (Farb and Boam, 2007). The high signal intensity for these AEs could be explained by the lack of symptom relief or the worsening of symptoms despite treatment with medications such as tirofiban, which may lead to clinical misdiagnosis. Bleeding from vascular access, hematoma at the cannulation site, and intracranial hemorrhage are well-established AEs of tirofiban use, aligning with those documented in the drug’s prescribing information. Based on the data from the FAERS and WHO-VigiAccess databases and the proportional imbalance analysis, these signals were statistically significant. However, while the statistical association is clear, the physiological relevance of these findings remains uncertain. Given the strength of these signals, it is essential to maintain close monitoring of patients for these AEs during the clinical course of tirofiban therapy.

#### 4.2.3 AEs related to blood and lymphatic system disorders

Our study identified Blood and lymphatic system disorders as a significant SOC for tirofiban associated AEs. Among these, thrombocytopenia emerged as the most prominent signal, although its underlying mechanism remains unclear. Muhammad (Rawala et al., 2020) reported a case of a 69-year-old female who received tirofiban before undergoing percutaneous coronary intervention. On the second day post-procedure, the patient developed ecchymosis and mild epistaxis. Further evaluation revealed a sharp decline in platelet count from 224 × 10^9^/L to 2 × 10^9^/L. Following a hematology consultation, the patient was treated with corticosteroids and transfused with two units of platelets. One week later, her platelet count increased to 442 × 10^9^/L. The thrombocytopenia was considered to be associated with tirofiban. Thrombocytopenia is defined as a platelet count of less than 100 × 10^9^/L or a reduction of more than 50% from baseline. The potential mechanisms underlying tirofiban-induced thrombocytopenia are as follows. Tirofiban may induce structural changes in platelet surface glycoprotein receptors, resulting in the formation of new antigenic determinants. These determinants can be recognized by antibodies to platelet glycoproteins in the blood. If cross-reactivity occurs between pre-existing antibodies and the new antigenic determinants, the platelets become coated with immunoglobulins and are cleared from circulation. Alternatively, these antigenic complexes may be recognized by the liver, leading to platelet sequestration and removal from circulation (Wang and Zou, 2023; Merlini et al., 2004; Rikken et al., 2023). The reported incidence of tirofiban-induced thrombocytopenia ranges from 0.4% to 5.6%, with severe cases potentially leading to increased mortality risk (Wang and Zou, 2023; Yi et al., 2018). Bleeding is typically the only symptom of thrombocytopenia, and upon confirmation of thrombocytopenia, it is recommended to discontinue tirofiban immediately. Subsequent adjustments to the use of medications such as aspirin, clopidogrel, and heparin should be based on whether bleeding-related complications are present. Platelet transfusion is required when the platelet count falls below 10 ×10^9^/L or in cases of severe bleeding. Consequently, routine blood testing, including platelet counts, hemoglobin levels, and erythrocyte counts, is recommended for all patients before administration, 6 h after the loading dose, and daily thereafter.

### 4.3 Other AE signals

In our analysis, we also detected unexpected and significant disproportionality signals for adverse events such as cerebral hemorrhage, cardiogenic shock, cardiac death, ventricular fibrillation, and the presence of drug-specific antibodies. All such novel and unforeseen adverse events warrant further investigation in future studies, and clinicians should remain vigilant during tirofiban administration in clinical practice.

### 4.4 TTO analysis

Understanding the timing of AE occurrence is crucial for early detection and intervention, as it helps identify specific risk windows and facilitates the prevention or early diagnosis of AEs. TTO analysis indicated that the majority of AEs occurred within 1 month after tirofiban administration, with relatively few events reported thereafter. Notably, bleeding and thrombocytopenia tended to manifest early after dosing, with median onset times of 0 days and 1 day, respectively, followed by a sharp decline in the incidence of AEs. Thrombocytopenia induced by tirofiban has been reported in the literature to typically occur within 24 h of treatment initiation, which is consistent with our findings (Merlini et al., 2004). However, in certain cases, delayed-onset thrombocytopenia may develop as late as 10 days after treatment (Clofent-Sanchez et al., 2007; Bosco et al., 2005). Additionally, we found that the AEs of tirofiban predominantly occurred within 30 days after administration. The Weibull distribution model showed that thrombocytopenia follows an early failure pattern, indicating a higher risk early in treatment, which diminishes over time. In contrast, bleeding events showed a random failure pattern, suggesting a consistent risk throughout therapy, regardless of treatment duration. Although these results may not reflect the actual TTO of a given population in clinical trials, our findings are close to those of previous studies. These results emphasize the need for early monitoring of thrombocytopenia, particularly in the first days of treatment, while ongoing vigilance is required for bleeding events across the entire treatment course. These findings underscore the importance of early monitoring for thrombocytopenia, particularly on the first day of treatment. Given that the median time to bleeding onset was 0 days, clinicians should closely observe patients for any signs of bleeding from the very beginning of therapy, including catheter-related bleeding. Special attention should be paid to severe events such as intracranial or cerebral hemorrhage.

### 4.5 Sensitivity analysis

The results of the reporter-stratified analysis appeared to be consistent with those of the overall AE analysis, which strengthens the reliability of our conclusions. The sex-stratified analysis showed that the signal for thrombocytopenia was significantly stronger among male patients. This may be related to the higher incidence of cardiovascular diseases in elderly men (Roth et al., 2021; Mensah et al., 2023), who are often prescribed multiple medications, thereby increasing the risk of drug-induced thrombocytopenia (Inna et al., 2018). In contrast, female patients exhibited a significantly higher risk of ischemic heart diseases (such as angina pectoris and coronary artery stenosis). This finding aligns with the established understanding of sex differences in coronary artery disease. Female patients are more prone to microvascular dysfunction and non-obstructive coronary artery disease, and their symptoms often present as atypical chest pain, which may lead to underdiagnosis or misdiagnosis in routine clinical settings (Noel et al., 2017).

The age-stratified analysis indicated that the overall safety profile of tirofiban was generally consistent across age groups, with most risk signals remaining stable between patients aged <65 and ≥65 years. This further supports the robustness of our conclusions. However, two notable heterogeneous signals warrant further investigation. Thrombosis in device showed a higher risk in younger patients, which may be associated with differences in the types of interventional procedures performed, device duration, or more active platelet physiology in this population. Additionally, hemoglobin decreased was reported more frequently among elderly patients, which likely reflects their higher baseline burden of comorbidities and greater frequency of clinical monitoring rather than a truelly drug-related effect. Moreover, the prevalence of anemia itself increases with age, often associated with chronic kidney disease, malnutrition, or chronic inflammatory conditions. Most cases in the elderly are mild (hemoglobin 10–12 g/dL) and rise progressively with age (Domenica and Irene, 2018). These findings suggest that, in clinical practice, particular attention should be paid to device-related thrombosis in younger patients, while regular monitoring of hemoglobin levels is recommended for elderly patients.

### 4.6 Clinical implications

This dual-database pharmacovigilance study offers several key insights for the safe use of tirofiban. The predominance of hemorrhage, thrombocytopenia, and intracranial bleeding confirms that bleeding and platelet complications remain its main safety concerns. According to our findings, thrombocytopenia associated with tirofiban typically occurs within the first 24 h after drug administration. In most cases, platelet counts gradually return to normal after discontinuation. Therefore, we recommend routine complete blood count monitoring before tirofiban administration and again at 6 h after the loading dose, including platelet count, hemoglobin, and hematocrit, followed by daily reassessment whenever possible during treatment. If thrombocytopenia is confirmed to be related to tirofiban, clinicians should discontinue the drug promptly. In patients with elderly, renal impairment or others at high risk of bleeding, dose adjustment should be considered. For example, in patients with a creatinine clearance ≤60 mL/min, a 50% of maintenance dose reduction is generally advised (Robert et al., 2023). If oral antiplatelet therapy is required as a bridging strategy after tirofiban use, imaging examinations should be repeated to rule out bleeding. In cases of minor bleeding (Bleeding Academic Research Consortium [BARC] types 1–2), discontinuation of therapy is not usually necessary; instead, close observation, symptomatic management, and treatment of the underlying condition are recommended (Chinese Stroke Society et al., 2019). For instance, proton pump inhibitors can be used for gastrointestinal bleeding. In the event of major bleeding, immediate discontinuation of tirofiban and prompt supportive management are advised, such as platelet transfusion.

### 4.7 Limitations

Several limitations should be acknowledged. First, the voluntary nature of reporting to both the FAERS and WHO-VigiAccess databases complicates the accurate estimation of AE incidence and prevalence, leading to underreporting. To address this limitation, alternative strategies have been explored, including linking spontaneous reporting data with external resources such as prescribing or dispensing databases. This approach can provide a more accurate context for background dose exposure patterns and improve the interpretation of safety signals (Okoli et al., 2021; Mokbel et al., 2022). Furthermore, the WHO-VigiAccess database lacks essential information, including reporter details, drug indications, outcomes, and TTO, making it difficult to validate some of these factors. Second, while this study used a more stringent selection process by focusing on PTs that were common across both databases and met four specific criteria, this may have led to the exclusion of certain new signals. Finally, due to the inherent limitations of the disproportionate analysis method, it is not possible to infer a causal relationship between the drug and the AE; it only provides an estimate of signal strength, which is statistically significant. Moreover, given the potential regional differences, the conclusions should be interpreted with caution. Therefore, prospective clinical trials are essential to establish definitive causal links.

## 5 Conclusion

In conclusion, this study provides a systematic and comprehensive analysis of tirofiban-related AEs by utilizing disproportionality analysis on data from the FAERS and WHO-VigiAccess databases. The AEs detected were largely consistent with those reported in the drug insert. Clinicians should be aware of potential off-label AEs, such as cardiac death and cerebral hemorrhage, and monitor patients closely during the first 1–2 days post-administration. Furthermore, we analyzed the median time to onset of these AEs, offering clinicians and pharmacists valuable insights to refine dosing strategies and enhance safety monitoring practices when using tirofiban. Despite the valuable insights provided, the exploratory nature of this study necessitates further research to establish causal relationships between these AEs and tirofiban.

## Data Availability

The datasets presented in this study can be found in online repositories. The names of the repository/repositories and accession number(s) can be found in the article/Supplementary Material.
