# Supplementary material for: Real-world safety of tirofiban: a disproportionality analysis using data from FAERS and WHO-VigiAccess
Source: Front Pharmacol. 2025 Dec 11;16:1713141. doi: 10.3389/fphar.2025.1713141 (PMC12738861; doi:10.3389/fphar.2025.1713141)
Supplement: Supplementary file 1 [file Supplementaryfile1.docx]

**Supplementary materials**

1. **Methods**

Disproportionation analysis is a data mining method, which is mainly used to evaluate the correlation between drugs and adverse reactions. The core principle is to use a 2 × 2 contingency table to compare the frequency of adverse events (AEs) observed in the exposed group and the non-exposed group, so as to quantify the association between drugs and adverse events. When the proportion of AEs in the exposed group exceeded that in the unexposed group, it was inferred that there was an association between drugs and specific AEs, indicating the presence of a disproportionation signal. After exceeding the threshold, the larger the signal value, the stronger the signal. In this study, we used four disproportional analysis methods: Reporting odds ratio (ROR), proportional reporting ratio (PRR), Bayesian confidence propagation neural network (BCPNN), and Muti-item Gamma Poisson Shrinker (MGPS). Specific data analysis methods are listed below:

Table S1. Two-by-two contingency table for disproportionality analysis.

| Item | Target AEs reported | Other AEs reported | Total |
| --- | --- | --- | --- |
| Tirofiban | a | b | a + b |
| Non-Tirofiban | c | d | c + d |
| Total | a + c | b + d | a + b + c + d |

Table S2. The principles of disproportionate measurement and the criteria for signal detection.

| Method | Calculation formula | ﻿Criteria |
| --- | --- | --- |
| ROR | $ROR=\frac{a / c}{b / d}$ | a ≥ 3  ROR ≥ 1  95%CI (lower limit) > 1 |
|  | $SE(lnROR)=\sqrt{\frac{1}{a}+\frac{1}{b}+\frac{1}{c}+\frac{1}{d}}$ |  |
|  | $95\%CI= e^{\ln\left( ROR \right)\pm1.96se}$ |  |
| PRR | $PRR=\frac{a / (a+b)}{c / (c+d)}$ | a ≥ 3  PRR ≥ 2  95%CI (lower limit) > 1 |
|  | $SE(lnPRR)=\sqrt{\frac{1}{a}-\frac{1}{a+b}+\frac{1}{c}-\frac{1}{c+d}}$ |  |
|  | $95\%CI= e^{\ln\left( PRR \right)\pm1.96se}$ |  |
|  | $\chi2 =\frac{{(ad-bc)}^{2}(a+b+c+d)}{( a+b)(a+c)(c+d)(b+d)}$ | a ≥ 3  PRR ≥ 2  $\chi2\geq4$ |
| BCPNN | IC=${log}_{2}\frac{p(x,y)}{p(x)p(y)}={log}_{2}\frac{a(a+b+c+d)}{(a+b)(a+c)}$ | IC025>0 |
|  | E(IC)=${log}_{2}\frac{(a+\gamma11)(a+b+c+d+\alpha)(a+b+c+d+\beta)}{（a+b+c+d+\gamma）(a+b+\alpha1)(a+c+\beta1)}$ |  |
|  | $V\left( IC \right)=\frac{1}{{(ln2)}^{2}}\{\left[ \frac{\left( a+b+c+d \right)-a+\gamma-\gamma11}{\left( a+\gamma11 \right)\left( 1+a+b+c+d+\gamma\right)} \right]+\left[ \frac{\left( a+b+c+d \right)-\left( a+b \right)+\alpha-\alpha1}{\left( a+b+\alpha1 \right)\left( 1+a+b+c+d+\alpha\right)} \right]+\left[ \frac{\left( a+b+c+d \right)-\left( a+c \right)+\beta-\beta1}{\left( a+c+\beta1 \right)\left( 1+a+b+c+d+\beta\right)} \right]\}$ |  |
|  | $\gamma=\gamma11\frac{(a+b+c+d+\alpha)(a+b+c+d+\beta)}{(a+b+\alpha1)(a+c+\beta1)}$ |  |
|  | *IC-2SD=E(IC)-2*$\sqrt{V(IC)}$  $\alpha1=\beta1=1；\alpha=\beta=2；\gamma11=1$ |  |
| MGPS | $EBGM=\frac{a(a+b+c+d)}{\left( a+c \right)(a+b)}$ | EBGM05>2 |
|  | $SE(lnEBGM)=\sqrt{\frac{1}{a}+\frac{1}{b}+\frac{1}{c}+\frac{1}{d}}$ |  |
|  | $95\%CI= e^{\ln\left( EBGM \right)\pm1.96se}$ |  |

a: count of reports with both specified drug and target adverse events; b: reports involving other adverse drug events with the specified drug; c: reports of target adverse drug events involving other drugs; d: reports encompassing other drugs and non-targeted adverse drug events. ROR: reporting odds ratio, PRR: proportional reporting ratio, BCPNN: Bayesian confidence propagation neural network, MGPS: Multi-item Gamma Poisson Shrinker , EBGM: empirical bayesian geometric mean, 95% CI: 95% confidence interval; N: number of reports; χ2: chi-squared; IC: information component; IC025: lower limit of 95% CI of the IC; E (IC): IC expectations; V(IC): variance of IC; EBGM05: lower limit of 95% CI of EBGM.

1. **Others**

**Table S3. Characteristics of AEs reports in FAERS database**

| **Characteristics** | ***FAERS(n=2421）***  ***N (%)*** |
| --- | --- |
| Reporter |  |
| Physician | 1495(61.75) |
| Other health-professional | 477(19.70) |
| Pharmacist | 311(12.85) |
| Consumer | 68( 2.81) |
| Not Specified | 70( 2.89) |
| Reporting countries |  |
| China | 1242(51.30) |
| United States of America | 379(15.65) |
| Germany | 180( 7.43) |
| Turkey | 166( 6.86) |
| Canada | 64( 2.64) |
| Not Specified | 59( 2.44) |
| Egypt | 57( 2.35) |
| Australia | 53( 2.19) |
| Italy | 42( 1.73) |
| Korea | 40( 1.65) |
| France | 29( 1.20) |
| Belgium | 20( 0.83) |
| Netherlands | 15( 0.62) |
| Switzerland | 13( 0.54) |
| India | 12( 0.50) |
| Jordan | 9( 0.37) |
| United Kingdom | 8( 0.33) |
| Lebanon | 7( 0.29) |
| Argentina | 6( 0.25) |
| Taiwan | 5( 0.21) |
| United Arab Emirates | 3( 0.12) |
| Estonia | 1( 0.04) |
| Pakistan | 1( 0.04) |
| Philippines | 1( 0.04) |
| Finland | 1( 0.04) |
| Colombia | 1( 0.04) |
| Croatia | 1( 0.04) |
| Norway | 1( 0.04) |
| Portugal | 1( 0.04) |
| Sweden | 1( 0.04) |
| Greece | 1( 0.04) |
| Spain | 1( 0.04) |
| Hungary | 1( 0.04) |
| Drug [indication](http://167.99.184.127/d/search.htm?s=indication) |  |
| Antiplatelet therapy | 1430(59.07) |
| Ischaemic stroke | 220( 9.09) |
| Off label use | 134( 5.53) |
| Not Specified | 113( 4.67) |
| Acute coronary syndrome | 107( 4.42) |
| Acute myocardial infarction | 95( 3.92) |
| Angina unstable | 60( 2.48) |
| Myocardial infarction | 37( 1.53) |
| Coronary angioplasty | 34( 1.40) |
| Angina unstable、Off label use | 15( 0.62) |
| Product used for unknown indication | 15( 0.62) |
| Acute myocardial infarction、Off label use | 13( 0.54) |
| Myocardial necrosis marker increased | 13( 0.54) |
| Cerebrovascular accident | 11( 0.45) |
| Acute myocardial infarction、Angina unstable | 7( 0.29) |
| Thrombosis | 6( 0.25) |
| Catheterisation cardiac | 5( 0.21) |
| Coronary arterial stent insertion | 5( 0.21) |
| Angina unstable、Myocardial infarction | 4( 0.17) |
| Anticoagulant therapy | 4( 0.17) |
| Cerebral haemorrhage | 4( 0.17) |
| Cerebral thrombosis | 4( 0.17) |
| Premedication | 4( 0.17) |
| Thrombosis prophylaxis | 4( 0.17) |
| Angioplasty | 3( 0.12) |
| Chest pain | 3( 0.12) |
| Coronary artery disease | 3( 0.12) |
| Coronary artery thrombosis | 3( 0.12) |
| Electrocardiogram ST segment depression、Myocardial necrosis marker increased | 3( 0.12) |
| Embolism | 3( 0.12) |
| Prophylaxis | 3( 0.12) |
| Angina pectoris | 2( 0.08) |
| Cerebral artery occlusion | 2( 0.08) |
| Electrocardiogram ST segment elevation | 2( 0.08) |
| Myocardial ischaemia | 2( 0.08) |
| Percutaneous coronary intervention | 2( 0.08) |
| Platelet aggregation inhibition | 2( 0.08) |
| Stent placement | 2( 0.08) |
| Thrombectomy | 2( 0.08) |
| Acute coronary syndrome、Angiocardiogram | 1( 0.04) |
| Acute myocardial infarction、Product used for unknown indication | 1( 0.04) |
| Angina pectoris、Angina unstable | 1( 0.04) |
| Angina pectoris、Electrocardiogram ST segment elevation | 1( 0.04) |
| Angina pectoris、Troponin increased | 1( 0.04) |
| Angina unstable、Enzyme abnormality | 1( 0.04) |
| Angina unstable、Myocardial necrosis marker increased | 1( 0.04) |
| Arterial stenosis | 1( 0.04) |
| Basilar artery occlusion | 1( 0.04) |
| Basilar artery thrombosis | 1( 0.04) |
| Cardiopulmonary bypass | 1( 0.04) |
| Cardiovascular event prophylaxis | 1( 0.04) |
| Catheterisation cardiac、Percutaneous coronary intervention | 1( 0.04) |
| Cerebral artery stenosis | 1( 0.04) |
| Cerebral infarction、Cerebrovascular accident | 1( 0.04) |
| Cerebrovascular accident、Carotid artery occlusion | 1( 0.04) |
| Coronary artery bypass | 1( 0.04) |
| Coronary artery surgery | 1( 0.04) |
| Electrocardiogram change | 1( 0.04) |
| Electrocardiogram ST segment depression | 1( 0.04) |
| Electrocardiogram ST segment elevation、Myocardial necrosis marker increased | 1( 0.04) |
| Electrocardiogram T wave inversion | 1( 0.04) |
| Electrocardiogram | 1( 0.04) |
| Haematoma | 1( 0.04) |
| Hyperlipidaemia | 1( 0.04) |
| Infarction | 1( 0.04) |
| Ischaemic cardiomyopathy | 1( 0.04) |
| Ischaemic heart disease prophylaxis | 1( 0.04) |
| Myocardial infarction、Acute coronary syndrome | 1( 0.04) |
| Platelet aggregation abnormal | 1( 0.04) |
| Platelet disorder | 1( 0.04) |
| Preoperative care | 1( 0.04) |
| Product use in unapproved indication | 1( 0.04) |
| Prophylaxis、Angiogram | 1( 0.04) |
| Thoracotomy | 1( 0.04) |
| Thrombocytopenia | 1( 0.04) |
| Thrombosis Acute coronary syndrome | 1( 0.04) |
| Thrombosis Percutaneous coronary intervention | 1( 0.04) |
| Vascular graft occlusion | 1( 0.04) |
| Vasodilation procedure | 1( 0.04) |
| Serious Reporter |  |
| Serious | 2115(87.36) |
| Non-Serious | 306(12.64) |
| Outcome |  |
| Life-Threatening | 290(11.98) |
| Hospitalization-Initial or Prolonged | 242(10.00) |
| Disability | 23( 0.95) |
| Death | 339(14.00) |
| Congenital Anomaly | 0( 0.00) |
| Required Intervention to Prevent Permanent Impairment/Damage | 32( 1.32) |
| Other | 1480(61.13) |

**Figure S1. Outcomes report distribution of AEs reports in the FAERS database.**


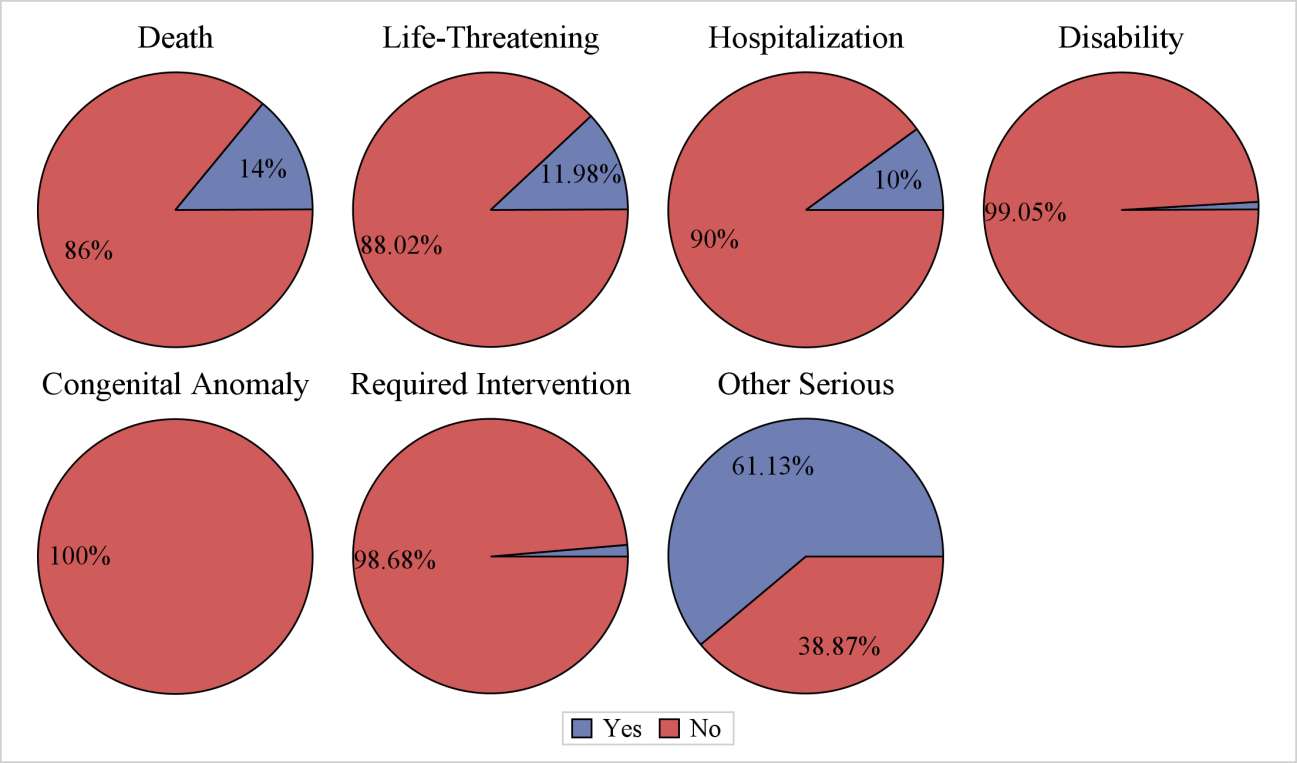


**Table S4. Signal strength of AEs at the System Organ Class (SOC) level in the FAERS database.**

| **System Organ Class (SOC)** | **SOC code** | **Case** | **ROR (95% CI)** | **PRR(χ2)** | **IC(IC025)** | **EBGM(EBGM05)** |
| --- | --- | --- | --- | --- | --- | --- |
| Cardiac disorders | 10007541 | 862 | 12.18(11.28,13.16) | 9.42(6656.83) | 3.23(3.11) | 9.41(8.72) |
| Vascular disorders | 10047065 | 547 | 8.55(7.80,9.36) | 7.36(3071.13) | 2.88(2.73) | 7.36(6.72) |
| Nervous system disorders | 10029205 | 414 | 1.45(1.31,1.61) | 1.40(51.79) | 0.49(0.33) | 1.40(1.26) |
| General disorders and administration site conditions | 10018065 | 386 | 0.59(0.53,0.65) | 0.63(98.31) | -0.66(-0.81) | 0.63(0.57) |
| Blood and lymphatic system disorders | 10005329 | 293 | 5.34(4.74,6.02) | 4.98(947.09) | 2.32(2.12) | 4.98(4.42) |
| Respiratory, thoracic and mediastinal disorders | 10038738 | 225 | 1.40(1.22,1.60) | 1.37(23.72) | 0.46(0.25) | 1.37(1.20) |
| Investigations | 10022891 | 183 | 0.85(0.73,0.98) | 0.86(4.78) | -0.23(-0.44) | 0.86(0.74) |
| Injury, poisoning and procedural complications | 10022117 | 153 | 0.40(0.34,0.46) | 0.42(135.32) | -1.25(-1.48) | 0.42(0.36) |
| Gastrointestinal disorders | 10017947 | 144 | 0.46(0.39,0.55) | 0.49(85.62) | -1.04(-1.28) | 0.49(0.41) |
| Surgical and medical procedures | 10042613 | 64 | 1.37(1.07,1.75) | 1.36(6.13) | 0.44(0.07) | 1.36(1.06) |
| Renal and urinary disorders | 10038359 | 52 | 0.78(0.59,1.03) | 0.78(3.16) | -0.35(-0.74) | 0.78(0.60) |
| Skin and subcutaneous tissue disorders | 10040785 | 39 | 0.20(0.14,0.27) | 0.21(124.92) | -2.27(-2.70) | 0.21(0.15) |
| Infections and infestations | 10021881 | 36 | 0.19(0.14,0.26) | 0.20(124.68) | -2.35(-2.79) | 0.20(0.14) |
| Product issues | 10077536 | 22 | 0.38(0.25,0.58) | 0.39(21.56) | -1.36(-1.93) | 0.39(0.26) |
| Eye disorders | 10015919 | 15 | 0.21(0.13,0.35) | 0.22(43.62) | -2.21(-2.86) | 0.22(0.13) |
| Musculoskeletal and connective tissue disorders | 10028395 | 12 | 0.06(0.04,0.11) | 0.07(165.74) | -3.91(-4.60) | 0.07(0.04) |
| Immune system disorders | 10021428 | 11 | 0.28(0.16,0.51) | 0.29(19.81) | -1.81(-2.55) | 0.29(0.16) |
| Hepatobiliary disorders | 10019805 | 9 | 0.28(0.15,0.54) | 0.28(16.69) | -1.83(-2.64) | 0.28(0.15) |
| Psychiatric disorders | 10037175 | 8 | 0.04(0.02,0.08) | 0.04(191.24) | -4.62(-5.42) | 0.04(0.02) |
| Metabolism and nutrition disorders | 10027433 | 3 | 0.04(0.01,0.12) | 0.04(71.27) | -4.66(-5.70) | 0.04(0.01) |
| Reproductive system and breast disorders | 10038604 | 3 | 0.10(0.03,0.30) | 0.10(25.46) | -3.37(-4.44) | 0.10(0.03) |
| Ear and labyrinth disorders | 10013993 | 2 | 0.13(0.03,0.53) | 0.13(11.43) | -2.92(-4.09) | 0.13(0.03) |
| Neoplasms benign, malignant and unspecified (incl cysts and polyps) | 10029104 | 2 | 0.02(0.01,0.09) | 0.02(89.78) | -5.51(-6.61) | 0.02(0.01) |
| Endocrine disorders | 10014698 | 2 | 0.22(0.06,0.90) | 0.23(5.35) | -2.15(-3.39) | 0.23(0.06) |

**Table S5. Signal strength of AEs at the System Organ Class (SOC) level in the WHO-VigiAccess database.**

| **System Organ Class (SOC)** | **SOC code** | **Case** | **ROR (95% CI)** | **PRR(χ2)** | **IC(IC025)** | **EBGM(EBGM05)** |
| --- | --- | --- | --- | --- | --- | --- |
| Blood and lymphatic system disorders | 10005329 | 1569 | 13.37(12.63,14.14) | 10.50(13783.0) | 3.39(3.30) | 10.49(9.92) |
| Gastrointestinal disorders | 10017947 | 838 | 1.28(1.19,1.38) | 1.25(45.58) | 0.32(0.21) | 1.25(1.16) |
| General disorders and administration site conditions | 10018065 | 722 | 0.47(0.43,0.51) | 0.53(387.51) | -0.93(-1.04) | 0.53(0.49) |
| Vascular disorders | 10047065 | 648 | 4.86(4.48,5.27) | 4.49(1794.99) | 2.17(2.04) | 4.49(4.14) |
| Cardiac disorders | 10007541 | 497 | 3.26(2.98,3.57) | 3.09(721.32) | 1.63(1.49) | 3.09(2.82) |
| Nervous system disorders | 10029205 | 493 | 0.69(0.63,0.75) | 0.71(64.53) | -0.49(-0.63) | 0.71(0.65) |
| Investigations | 10022891 | 442 | 1.04(0.94,1.14) | 1.03(0.52) | 0.05(-0.09) | 1.03(0.94) |
| Respiratory, thoracic and mediastinal disorders | 10038738 | 404 | 1.38(1.25,1.53) | 1.36(39.71) | 0.44(0.29) | 1.36(1.23) |
| Skin and subcutaneous tissue disorders | 10040785 | 329 | 0.53(0.47,0.59) | 0.55(132.61) | -0.86(-1.02) | 0.55(0.49) |
| Injury, poisoning and procedural complications | 10022117 | 264 | 0.59(0.52,0.67) | 0.61(71.10) | -0.72(-0.90) | 0.61(0.54) |
| Renal and urinary disorders | 10038359 | 262 | 2.47(2.19,2.80) | 2.42(221.11) | 1.27(1.08) | 2.42(2.14) |
| Immune system disorders | 10021428 | 63 | 0.74(0.58,0.95) | 0.74(5.80) | -0.43(-0.79) | 0.74(0.58) |
| Eye disorders | 10015919 | 48 | 0.41(0.31,0.54) | 0.41(41.31) | -1.28(-1.68) | 0.41(0.31) |
| Musculoskeletal and connective tissue disorders | 10028395 | 47 | 0.13(0.09,0.17) | 0.13(283.58) | -2.92(-3.31) | 0.13(0.10) |
| Infections and infestations | 10021881 | 35 | 0.12(0.09,0.17) | 0.13(214.48) | -2.95(-3.40) | 0.13(0.09) |
| Psychiatric disorders | 10037175 | 35 | 0.10(0.07,0.15) | 0.11(267.69) | -3.20(-3.64) | 0.11(0.08) |
| Surgical and medical procedures | 10042613 | 21 | 0.38(0.25,0.58) | 0.38(21.10) | -1.39(-1.96) | 0.38(0.25) |
| Metabolism and nutrition disorders | 10027433 | 15 | 0.12(0.07,0.20) | 0.12(94.67) | -3.01(-3.65) | 0.12(0.07) |
| Hepatobiliary disorders | 10019805 | 12 | 0.22(0.13,0.39) | 0.22(32.88) | -2.17(-2.88) | 0.22(0.13) |
| Product issues | 10077536 | 8 | 0.13(0.07,0.26) | 0.13(46.28) | -2.93(-3.74) | 0.13(0.07) |
| Ear and labyrinth disorders | 10013993 | 7 | 0.19(0.09,0.40) | 0.19(23.98) | -2.38(-3.25) | 0.19(0.09) |
| Reproductive system and breast disorders | 10038604 | 7 | 0.10(0.05,0.20) | 0.10(59.53) | -3.37(-4.21) | 0.10(0.05) |
| Neoplasms benign, malignant and unspecified (incl cysts and polyps) | 10029104 | 2 | 0.02(0.01,0.08) | 0.02(91.80) | -5.56(-6.66) | 0.02(0.01) |
| Congenital, familial and genetic disorders | 10010331 | 1 | 0.09(0.01,0.62) | 0.09(9.62) | -3.53(-4.69) | 0.09(0.01) |
| Pregnancy, puerperium and perinatal conditions | 10036585 | 1 | 0.05(0.01,0.39) | 0.05(16.30) | -4.19(-5.30) | 0.05(0.01) |

**Table S6. Signal strength of tirofiban-related AEs at the preferred term (PT) level in the FAERS database**

| **System Organ Class (SOC)** | **Preferred Term (PT)** | **PT code** | **Case reports** | **ROR (95% CI)** | **PRR(χ2)** | **IC(IC025)** | **EBGM(EBGM05)** |
| --- | --- | --- | --- | --- | --- | --- | --- |
| Vascular disorders | Haemorrhage | 10055798 | 370 | 71.12(63.84,79.23) | 63.68(22774.5) | 5.99(5.60) | 63.43(56.94) |
| Blood and lymphatic system disorders | Thrombocytopenia | 10043554 | 248 | 42.79(37.60,48.70) | 39.82(9379.40) | 5.31(4.91) | 39.72(34.91) |
| Cardiac disorders | Myocardial infarction | 10028596 | 207 | 20.95(18.20,24.11) | 19.76(3693.82) | 4.30(3.97) | 19.74(17.15) |
| Cardiac disorders | Angina pectoris | 10002383 | 181 | 113.77(97.91,132.19) | 107.91(19051.5) | 6.74(5.86) | 107.19(92.25) |
| Nervous system disorders | Haemorrhage intracranial | 10018985 | 176 | 211.77(181.81,246.66) | 201.13(34618.0) | 7.63(6.33) | 198.63(170.53) |
| General disorders and administration site conditions | Death | 10011906 | 132 | 2.81(2.36,3.34) | 2.74(147.70) | 1.45(1.18) | 2.74(2.30) |
| Cardiac disorders | Cardiac failure acute | 10007556 | 117 | 337.71(280.35,406.81) | 326.42(37196.1) | 8.32(6.16) | 319.86(265.53) |
| Nervous system disorders | Cerebral haemorrhage | 10008111 | 96 | 48.33(39.44,59.21) | 47.02(4314.07) | 5.55(4.69) | 46.89(38.27) |
| Respiratory, thoracic and mediastinal disorders | Acute respiratory failure | 10001053 | 95 | 92.75(75.60,113.79) | 90.25(8339.87) | 6.49(5.24) | 89.74(73.15) |
| Cardiac disorders | Cardiac failure | 10007554 | 80 | 17.95(14.38,22.41) | 17.56(1249.56) | 4.13(3.54) | 17.54(14.05) |
| General disorders and administration site conditions | Vascular stent thrombosis | 10063934 | 55 | 502.58(383.49,658.66) | 494.67(26278.9) | 8.91(5.26) | 479.75(366.07) |
| Vascular disorders | Infarction | 10061216 | 53 | 132.25(100.72,173.66) | 130.26(6743.59) | 7.01(4.86) | 129.21(98.40) |
| Cardiac disorders | Cardiogenic shock | 10007625 | 38 | 49.84(36.18,68.64) | 49.30(1793.13) | 5.62(3.99) | 49.15(35.69) |
| Investigations | Haemoglobin decreased | 10018884 | 32 | 5.43(3.84,7.70) | 5.39(114.64) | 2.43(1.75) | 5.39(3.81) |
| General disorders and administration site conditions | Cardiac death | 10049993 | 31 | 453.85(317.10,649.56) | 449.82(13500.3) | 8.77(4.38) | 437.46(305.65) |
| Gastrointestinal disorders | Gastrointestinal haemorrhage | 10017955 | 29 | 5.89(4.09,8.49) | 5.85(116.69) | 2.55(1.80) | 5.85(4.06) |
| Cardiac disorders | Arrhythmia | 10003119 | 28 | 10.25(7.07,14.87) | 10.18(231.81) | 3.35(2.41) | 10.17(7.01) |
| Cardiac disorders | Acute myocardial infarction | 10000891 | 25 | 14.43(9.73,21.38) | 14.33(309.89) | 3.84(2.67) | 14.32(9.66) |
| Cardiac disorders | Coronary artery stenosis | 10011089 | 24 | 80.28(53.68,120.06) | 79.74(1856.83) | 6.31(3.68) | 79.34(53.05) |
| Vascular disorders | Haematoma | 10018852 | 24 | 15.94(10.66,23.81) | 15.83(333.32) | 3.98(2.73) | 15.82(10.59) |
| Renal and urinary disorders | Haematuria | 10018867 | 23 | 11.62(7.71,17.51) | 11.55(221.57) | 3.53(2.41) | 11.54(7.66) |
| Surgical and medical procedures | Coronary revascularisation | 10049887 | 21 | 2640.80(1662.24,4195.43) | 2624.90(47265.5) | 11.14(3.79) | 2252.59(1417.88) |
| Surgical and medical procedures | Craniectomy | 10052937 | 21 | 6844.52(4100.70,11424.3) | 6803.31(99979.4) | 12.22(3.75) | 4762.62(2853.39) |
| Respiratory, thoracic and mediastinal disorders | Haemoptysis | 10018964 | 20 | 12.55(8.09,19.49) | 12.49(211.30) | 3.64(2.38) | 12.48(8.04) |
| Respiratory, thoracic and mediastinal disorders | Pulmonary alveolar haemorrhage | 10037313 | 19 | 62.68(39.90,98.47) | 62.34(1142.43) | 5.96(3.29) | 62.10(39.53) |
| Vascular disorders | Thrombosis | 10043607 | 18 | 3.91(2.46,6.21) | 3.90(38.78) | 1.96(1.09) | 3.89(2.45) |
| Product issues | Thrombosis in device | 10062546 | 18 | 90.34(56.78,143.75) | 89.88(1573.28) | 6.48(3.32) | 89.38(56.17) |
| Injury, poisoning and procedural complications | Reocclusion | 10038563 | 17 | 4757.66(2765.15,8185.92) | 4734.47(61969.8) | 11.83(3.40) | 3647.05(2119.66) |
| Vascular disorders | Embolism | 10061169 | 17 | 35.39(21.97,57.03) | 35.23(564.15) | 5.14(2.92) | 35.15(21.81) |
| Cardiac disorders | Ventricular arrhythmia | 10047281 | 16 | 77.58(47.41,126.92) | 77.22(1198.04) | 6.26(3.11) | 76.86(46.97) |
| General disorders and administration site conditions | Catheter site haemorrhage | 10051099 | 15 | 110.15(66.22,183.23) | 109.68(1604.32) | 6.77(3.09) | 108.93(65.49) |
| Skin and subcutaneous tissue disorders | Petechiae | 10034754 | 14 | 23.96(14.17,40.51) | 23.86(306.27) | 4.57(2.49) | 23.83(14.09) |
| Cardiac disorders | Ventricular fibrillation | 10047290 | 14 | 22.04(13.04,37.27) | 21.96(279.72) | 4.45(2.45) | 21.93(12.97) |
| Injury, poisoning and procedural complications | Post procedural complication | 10058046 | 14 | 12.61(7.46,21.31) | 12.56(148.90) | 3.65(2.08) | 12.55(7.42) |
| Gastrointestinal disorders | Gingival bleeding | 10018276 | 13 | 16.78(9.73,28.94) | 16.72(192.02) | 4.06(2.20) | 16.71(9.69) |
| Gastrointestinal disorders | Retroperitoneal haemorrhage | 10038980 | 13 | 88.48(51.25,152.76) | 88.15(1114.00) | 6.45(2.83) | 87.67(50.78) |
| Investigations | Haematocrit decreased | 10018838 | 11 | 9.37(5.18,16.93) | 9.34(81.91) | 3.22(1.63) | 9.34(5.16) |
| Injury, poisoning and procedural complications | Restenosis | 10082493 | 11 | 5004.88(2539.85,9862.31) | 4989.09(41739.8) | 11.89(2.65) | 3796.29(1926.52) |
| Investigations | Drug specific antibody present | 10013745 | 10 | 27.71(14.89,51.58) | 27.64(256.31) | 4.79(2.14) | 27.59(14.82) |
| Respiratory, thoracic and mediastinal disorders | Pulmonary haemorrhage | 10037394 | 10 | 21.86(11.74,40.67) | 21.80(198.18) | 4.44(2.04) | 21.77(11.70) |
| Gastrointestinal disorders | Upper gastrointestinal haemorrhage | 10046274 | 10 | 10.25(5.51,19.07) | 10.22(83.19) | 3.35(1.60) | 10.22(5.49) |
| Investigations | Ejection fraction decreased | 10050528 | 10 | 11.33(6.09,21.08) | 11.30(93.84) | 3.50(1.67) | 11.29(6.07) |
| Cardiac disorders | Acute coronary syndrome | 10051592 | 10 | 20.37(10.95,37.91) | 20.32(183.46) | 4.34(2.01) | 20.29(10.90) |
| Nervous system disorders | Ischaemic stroke | 10061256 | 10 | 9.82(5.28,18.27) | 9.79(78.91) | 3.29(1.57) | 9.79(5.26) |
| Gastrointestinal disorders | Haematemesis | 10018830 | 9 | 6.17(3.21,11.86) | 6.15(38.84) | 2.62(1.11) | 6.15(3.20) |
| Nervous system disorders | Subarachnoid haemorrhage | 10042316 | 9 | 15.35(7.98,29.53) | 15.31(120.28) | 3.94(1.74) | 15.30(7.95) |
| General disorders and administration site conditions | Sudden cardiac death | 10049418 | 9 | 43.79(22.74,84.30) | 43.68(374.30) | 5.44(2.14) | 43.56(22.63) |
| Vascular disorders | Shock haemorrhagic | 10049771 | 9 | 20.19(10.49,38.85) | 20.14(163.52) | 4.33(1.87) | 20.12(10.45) |
| Injury, poisoning and procedural complications | Post procedural haemorrhage | 10051077 | 9 | 13.35(6.94,25.69) | 13.32(102.50) | 3.73(1.66) | 13.31(6.92) |
| Cardiac disorders | Cardiac ventricular thrombosis | 10053994 | 9 | 36.09(18.75,69.47) | 36.00(305.55) | 5.17(2.08) | 35.92(18.66) |
| Nervous system disorders | Intracranial haematoma | 10059491 | 9 | 276.52(142.95,534.92) | 275.81(2422.26) | 8.08(2.35) | 271.12(140.15) |
| Cardiac disorders | Coronary artery thrombosis | 10011091 | 8 | 66.54(33.20,133.35) | 66.39(513.08) | 6.05(2.04) | 66.11(32.99) |
| General disorders and administration site conditions | Injection site haematoma | 10022066 | 8 | 8.93(4.46,17.86) | 8.91(56.14) | 3.15(1.28) | 8.90(4.45) |
| Cardiac disorders | Ventricular tachycardia | 10047302 | 8 | 8.50(4.25,17.02) | 8.49(52.82) | 3.08(1.25) | 8.48(4.24) |
| Cardiac disorders | Pulseless electrical activity | 10058151 | 8 | 28.69(14.32,57.44) | 28.62(212.89) | 4.84(1.85) | 28.57(14.27) |
| Blood and lymphatic system disorders | Haemolysis | 10018910 | 7 | 15.99(7.61,33.57) | 15.96(98.05) | 3.99(1.45) | 15.94(7.59) |
| Cardiac disorders | Cardiac tamponade | 10007610 | 6 | 22.09(9.91,49.24) | 22.06(120.46) | 4.46(1.37) | 22.03(9.88) |
| Surgical and medical procedures | Coronary artery bypass | 10011077 | 6 | 17.02(7.64,37.92) | 16.99(90.21) | 4.09(1.28) | 16.97(7.62) |
| Injury, poisoning and procedural complications | Vascular pseudoaneurysm | 10048975 | 6 | 61.59(27.61,137.41) | 61.49(355.66) | 5.94(1.58) | 61.26(27.46) |
| Vascular disorders | Haemodynamic instability | 10052076 | 6 | 14.68(6.59,32.71) | 14.66(76.28) | 3.87(1.22) | 14.64(6.57) |
| Injury, poisoning and procedural complications | Vascular access site haemorrhage | 10077643 | 6 | 883.43(388.02,2011.38) | 881.91(5001.61) | 9.71(1.67) | 835.55(366.99) |
| Investigations | Activated partial thromboplastin time prolonged | 10000636 | 5 | 16.59(6.90,39.91) | 16.57(73.08) | 4.05(1.02) | 16.55(6.88) |
| Respiratory, thoracic and mediastinal disorders | Acute pulmonary oedema | 10001029 | 5 | 15.47(6.43,37.22) | 15.45(67.53) | 3.95(1.00) | 15.44(6.42) |
| Blood and lymphatic system disorders | Coagulopathy | 10009802 | 5 | 5.23(2.17,12.57) | 5.22(17.06) | 2.38(0.44) | 5.22(2.17) |
| Blood and lymphatic system disorders | Disseminated intravascular coagulation | 10013442 | 5 | 6.10(2.54,14.66) | 6.09(21.26) | 2.61(0.54) | 6.09(2.53) |
| Skin and subcutaneous tissue disorders | Ecchymosis | 10014080 | 5 | 12.50(5.20,30.07) | 12.49(52.80) | 3.64(0.92) | 12.48(5.19) |
| Nervous system disorders | Haemorrhagic stroke | 10019016 | 5 | 11.02(4.58,26.50) | 11.00(45.45) | 3.46(0.86) | 11.00(4.57) |
| Cardiac disorders | Left ventricular failure | 10024119 | 5 | 25.06(10.42,60.28) | 25.02(115.14) | 4.64(1.14) | 24.98(10.39) |
| General disorders and administration site conditions | Catheter site haematoma | 10055662 | 5 | 308.08(127.07,746.94) | 307.64(1499.19) | 8.24(1.37) | 301.81(124.49) |
| Investigations | Troponin T increased | 10058269 | 5 | 71.41(29.65,172.02) | 71.31(345.09) | 6.15(1.30) | 71.00(29.47) |
| Cardiac disorders | Angina unstable | 10002388 | 4 | 9.95(3.73,26.52) | 9.94(32.13) | 3.31(0.54) | 9.93(3.72) |
| Injury, poisoning and procedural complications | Brain herniation | 10006126 | 4 | 23.92(8.96,63.80) | 23.89(87.60) | 4.58(0.81) | 23.85(8.94) |
| Cardiac disorders | Bundle branch block left | 10006580 | 4 | 15.94(5.97,42.50) | 15.92(55.87) | 3.99(0.71) | 15.90(5.96) |
| Nervous system disorders | Carotid artery stenosis | 10007687 | 4 | 19.06(7.15,50.84) | 19.04(68.29) | 4.25(0.75) | 19.02(7.13) |
| Investigations | Electrocardiogram ST segment elevation | 10014392 | 4 | 20.68(7.75,55.16) | 20.66(74.73) | 4.37(0.77) | 20.63(7.73) |
| Vascular disorders | Hypovolaemic shock | 10021138 | 4 | 15.01(5.63,40.04) | 15.00(52.21) | 3.91(0.69) | 14.98(5.62) |
| Respiratory, thoracic and mediastinal disorders | Lung infiltration | 10025102 | 4 | 7.45(2.79,19.86) | 7.44(22.29) | 2.89(0.41) | 7.44(2.79) |
| Nervous system disorders | Brain oedema | 10048962 | 4 | 5.66(2.12,15.08) | 5.65(15.31) | 2.50(0.26) | 5.65(2.12) |
| General disorders and administration site conditions | Puncture site haemorrhage | 10051101 | 4 | 99.33(37.14,265.61) | 99.21(386.49) | 6.62(0.97) | 98.60(36.87) |
| Injury, poisoning and procedural complications | Coronary artery restenosis | 10056489 | 4 | 106.84(39.94,285.77) | 106.72(416.12) | 6.73(0.97) | 106.01(39.63) |
| Injury, poisoning and procedural complications | Procedural complication | 10057765 | 4 | 8.79(3.30,23.44) | 8.78(27.57) | 3.13(0.49) | 8.78(3.29) |
| Nervous system disorders | Capsular warning syndrome | 10067744 | 4 | 10595.1(2988.52,37562.3) | 10582.9(25394.2) | 12.63(0.76) | 6350.15(1791.17) |
| General disorders and administration site conditions | Device embolisation | 10074896 | 4 | 236.32(88.00,634.61) | 236.05(922.51) | 7.86(0.99) | 232.61(86.62) |
| General disorders and administration site conditions | Vascular stent occlusion | 10077143 | 4 | 176.09(65.70,472.01) | 175.89(687.98) | 7.44(0.99) | 173.98(64.91) |
| Nervous system disorders | Brain stem infarction | 10006147 | 3 | 47.95(15.43,149.00) | 47.91(137.38) | 5.58(0.47) | 47.77(15.37) |
| Investigations | Electrocardiogram ST segment depression | 10014391 | 3 | 23.05(7.42,71.56) | 23.03(63.13) | 4.52(0.38) | 23.00(7.41) |
| Respiratory, thoracic and mediastinal disorders | Haemothorax | 10019027 | 3 | 17.01(5.48,52.80) | 17.00(45.12) | 4.09(0.32) | 16.98(5.47) |
| Nervous system disorders | Intraventricular haemorrhage | 10022840 | 3 | 20.38(6.56,63.26) | 20.36(55.16) | 4.35(0.36) | 20.34(6.55) |
| Vascular disorders | Vascular rupture | 10053649 | 3 | 33.47(10.78,103.96) | 33.44(94.22) | 5.06(0.43) | 33.38(10.75) |
| Injury, poisoning and procedural complications | Cardiac procedure complication | 10057461 | 3 | 81.62(26.23,253.92) | 81.55(237.46) | 6.34(0.50) | 81.13(26.08) |
| Vascular disorders | Peripheral artery occlusion | 10057525 | 3 | 30.57(9.84,94.94) | 30.55(85.58) | 4.93(0.42) | 30.49(9.82) |
| Gastrointestinal disorders | Retroperitoneal haematoma | 10058360 | 3 | 23.65(7.62,73.44) | 23.63(64.94) | 4.56(0.38) | 23.60(7.60) |
| Nervous system disorders | Embolic cerebral infarction | 10060839 | 3 | 79.31(25.49,246.72) | 79.24(230.61) | 6.30(0.50) | 78.85(25.35) |
| General disorders and administration site conditions | Mucosal haemorrhage | 10061298 | 3 | 45.44(14.62,141.18) | 45.40(129.89) | 5.50(0.46) | 45.27(14.57) |
| General disorders and administration site conditions | Disease complication | 10067671 | 3 | 11.41(3.68,35.42) | 11.40(28.46) | 3.51(0.22) | 11.40(3.67) |

**Table S7. Signal strength of tirofiban-related AEs at the PT level in the WHO-VigiAccess database**

| **System Organ Class (SOC)** | **Preferred Term (PT)** | **PT code** | **Case reports** | **ROR (95% CI)** | **PRR(χ2)** | **IC(IC025)** | **EBGM(EBGM05)** |
| --- | --- | --- | --- | --- | --- | --- | --- |
| Blood and lymphatic system disorders | Thrombocytopenia | 10043554 | 1391 | 89.61(84.47,95.06) | 71.40(96442.6) | 6.15(6.00) | 71.11(67.04) |
| Vascular disorders | Haemorrhage | 10055798 | 223 | 28.48(24.92,32.55) | 27.58(5709.44) | 4.78(4.43) | 27.53(24.09) |
| Renal and urinary disorders | Haematuria | 10018867 | 190 | 44.02(38.10,50.86) | 42.81(7745.08) | 5.42(4.92) | 42.71(36.97) |
| Gastrointestinal disorders | Gingival bleeding | 10018276 | 157 | 105.86(90.32,124.07) | 103.43(15834.9) | 6.68(5.73) | 102.82(87.73) |
| Vascular disorders | Haematoma | 10018852 | 147 | 40.25(34.18,47.41) | 39.40(5491.92) | 5.30(4.72) | 39.31(33.38) |
| Gastrointestinal disorders | Gastrointestinal haemorrhage | 10017955 | 138 | 16.28(13.76,19.28) | 15.97(1937.50) | 4.00(3.60) | 15.96(13.48) |
| Investigations | Platelet count decreased | 10035528 | 116 | 13.98(11.64,16.80) | 13.76(1373.40) | 3.78(3.36) | 13.75(11.44) |
| Nervous system disorders | Cerebral haemorrhage | 10008111 | 105 | 29.47(24.30,35.74) | 29.03(2838.33) | 4.86(4.24) | 28.98(23.90) |
| Cardiac disorders | Myocardial infarction | 10028596 | 89 | 7.49(6.08,9.24) | 7.41(493.92) | 2.89(2.48) | 7.40(6.01) |
| Respiratory, thoracic and mediastinal disorders | Haemoptysis | 10018964 | 88 | 36.81(29.82,45.43) | 36.34(3019.10) | 5.18(4.39) | 36.27(29.38) |
| Investigations | Haemoglobin decreased | 10018884 | 80 | 10.12(8.12,12.62) | 10.01(649.45) | 3.32(2.85) | 10.01(8.03) |
| Nervous system disorders | Haemorrhage intracranial | 10018985 | 77 | 56.01(44.73,70.14) | 55.39(4099.89) | 5.79(4.70) | 55.21(44.09) |
| Respiratory, thoracic and mediastinal disorders | Epistaxis | 10015090 | 74 | 8.24(6.56,10.37) | 8.16(465.67) | 3.03(2.56) | 8.16(6.49) |
| Vascular disorders | Hypotension | 10021097 | 71 | 2.93(2.32,3.70) | 2.91(89.44) | 1.54(1.16) | 2.91(2.30) |
| Gastrointestinal disorders | Haematemesis | 10018830 | 71 | 25.69(20.33,32.46) | 25.43(1664.53) | 4.67(3.90) | 25.39(20.10) |
| Gastrointestinal disorders | Upper gastrointestinal haemorrhage | 10046274 | 57 | 30.77(23.70,39.94) | 30.52(1624.98) | 4.93(3.96) | 30.47(23.47) |
| Blood and lymphatic system disorders | Anaemia | 10002034 | 57 | 2.77(2.14,3.60) | 2.76(64.13) | 1.46(1.04) | 2.76(2.13) |
| Respiratory, thoracic and mediastinal disorders | Pulmonary haemorrhage | 10037394 | 50 | 81.10(61.36,107.18) | 80.51(3908.31) | 6.32(4.57) | 80.14(60.64) |
| Gastrointestinal disorders | Mouth haemorrhage | 10028024 | 45 | 71.61(53.38,96.06) | 71.14(3099.59) | 6.15(4.39) | 70.86(52.82) |
| Gastrointestinal disorders | Retroperitoneal haemorrhage | 10038980 | 40 | 129.26(94.62,176.58) | 128.50(5023.60) | 7.00(4.51) | 127.57(93.38) |
| General disorders and administration site conditions | Injection site haemorrhage | 10022067 | 39 | 7.52(5.49,10.30) | 7.48(219.09) | 2.90(2.23) | 7.48(5.46) |
| Skin and subcutaneous tissue disorders | Ecchymosis | 10014080 | 39 | 29.92(21.84,41.00) | 29.76(1082.15) | 4.89(3.65) | 29.71(21.68) |
| Cardiac disorders | Cardiac arrest | 10007515 | 38 | 5.38(3.91,7.40) | 5.35(134.61) | 2.42(1.80) | 5.35(3.89) |
| Skin and subcutaneous tissue disorders | Petechiae | 10034754 | 37 | 17.75(12.85,24.52) | 17.66(581.07) | 4.14(3.15) | 17.64(12.77) |
| Gastrointestinal disorders | Melaena | 10027141 | 34 | 10.10(7.21,14.15) | 10.06(277.24) | 3.33(2.51) | 10.05(7.17) |
| Investigations | Haematocrit decreased | 10018838 | 32 | 19.31(13.64,27.33) | 19.22(552.24) | 4.26(3.13) | 19.20(13.56) |
| Cardiac disorders | Cardiogenic shock | 10007625 | 30 | 37.88(26.45,54.24) | 37.72(1070.11) | 5.23(3.59) | 37.64(26.28) |
| Blood and lymphatic system disorders | Coagulopathy | 10009802 | 26 | 13.73(9.34,20.18) | 13.68(305.37) | 3.77(2.66) | 13.67(9.30) |
| Cardiac disorders | Angina pectoris | 10002383 | 26 | 8.73(5.94,12.83) | 8.70(177.13) | 3.12(2.20) | 8.69(5.91) |
| Vascular disorders | Thrombosis | 10043607 | 25 | 4.23(2.86,6.26) | 4.22(61.39) | 2.08(1.34) | 4.22(2.85) |
| Cardiac disorders | Cardiac failure | 10007554 | 25 | 4.72(3.19,6.99) | 4.71(73.02) | 2.23(1.47) | 4.71(3.18) |
| Gastrointestinal disorders | Haematochezia | 10018836 | 25 | 5.86(3.96,8.68) | 5.84(100.35) | 2.55(1.73) | 5.84(3.94) |
| Nervous system disorders | Subarachnoid haemorrhage | 10042316 | 24 | 27.62(18.49,41.25) | 27.52(612.54) | 4.78(3.16) | 27.48(18.40) |
| Skin and subcutaneous tissue disorders | Purpura | 10037549 | 24 | 9.28(6.22,13.86) | 9.25(176.64) | 3.21(2.22) | 9.25(6.19) |
| Nervous system disorders | Coma | 10010071 | 22 | 5.06(3.33,7.68) | 5.04(71.34) | 2.33(1.50) | 5.04(3.32) |
| General disorders and administration site conditions | Cardiac death | 10049993 | 20 | 288.73(185.49,449.45) | 287.88(5624.77) | 8.15(3.66) | 283.22(181.94) |
| Respiratory, thoracic and mediastinal disorders | Pulmonary alveolar haemorrhage | 10037313 | 20 | 62.36(40.17,96.79) | 62.17(1199.58) | 5.95(3.36) | 61.96(39.91) |
| Injury, poisoning and procedural complications | Product administration error | 10081576 | 20 | 4.64(2.99,7.20) | 4.63(56.91) | 2.21(1.35) | 4.63(2.98) |
| Vascular disorders | Circulatory collapse | 10009192 | 19 | 7.75(4.94,12.16) | 7.73(111.40) | 2.95(1.89) | 7.73(4.93) |
| Gastrointestinal disorders | Faeces discoloured | 10016100 | 19 | 8.72(5.56,13.68) | 8.70(129.46) | 3.12(2.00) | 8.70(5.54) |
| Injury, poisoning and procedural complications | Post procedural haemorrhage | 10051077 | 18 | 20.20(12.72,32.09) | 20.15(327.28) | 4.33(2.66) | 20.13(12.67) |
| Gastrointestinal disorders | Rectal haemorrhage | 10038063 | 18 | 4.75(2.99,7.54) | 4.74(53.05) | 2.24(1.32) | 4.73(2.98) |
| Vascular disorders | Aneurysm | 10002329 | 17 | 47.24(29.33,76.09) | 47.12(765.44) | 5.55(3.04) | 47.00(29.18) |
| Vascular disorders | Shock haemorrhagic | 10049771 | 17 | 28.16(17.49,45.34) | 28.09(443.51) | 4.81(2.80) | 28.05(17.42) |
| Cardiac disorders | Cardiac tamponade | 10007610 | 17 | 60.62(37.63,97.66) | 60.48(990.96) | 5.91(3.13) | 60.27(37.41) |
| Cardiac disorders | Acute myocardial infarction | 10000891 | 17 | 8.68(5.39,13.97) | 8.66(115.11) | 3.11(1.92) | 8.65(5.38) |
| Skin and subcutaneous tissue disorders | Haemorrhage subcutaneous | 10018999 | 17 | 38.58(23.96,62.13) | 38.49(619.38) | 5.26(2.96) | 38.40(23.85) |
| Cardiac disorders | Ventricular fibrillation | 10047290 | 17 | 17.56(10.91,28.28) | 17.52(264.60) | 4.13(2.51) | 17.50(10.87) |
| Investigations | Prothrombin time prolonged | 10037063 | 16 | 19.27(11.79,31.48) | 19.22(276.15) | 4.26(2.51) | 19.20(11.75) |
| Nervous system disorders | Hemiparesis | 10019465 | 16 | 9.97(6.10,16.28) | 9.95(128.71) | 3.31(2.00) | 9.94(6.09) |
| Gastrointestinal disorders | Lower gastrointestinal haemorrhage | 10050953 | 16 | 22.49(13.77,36.75) | 22.44(327.40) | 4.49(2.61) | 22.41(13.72) |
| Vascular disorders | Shock | 10040560 | 15 | 7.85(4.73,13.03) | 7.84(89.46) | 2.97(1.73) | 7.83(4.72) |
| General disorders and administration site conditions | Catheter site haemorrhage | 10051099 | 15 | 92.80(55.84,154.23) | 92.60(1352.00) | 6.53(3.06) | 92.12(55.43) |
| Gastrointestinal disorders | Gastric haemorrhage | 10017788 | 15 | 15.96(9.61,26.49) | 15.92(209.64) | 3.99(2.32) | 15.91(9.58) |
| Respiratory, thoracic and mediastinal disorders | Pulmonary oedema | 10037423 | 15 | 4.57(2.75,7.58) | 4.56(41.68) | 2.19(1.18) | 4.56(2.75) |
| Cardiac disorders | Coronary artery occlusion | 10011086 | 13 | 15.23(8.84,26.25) | 15.20(172.38) | 3.93(2.14) | 15.19(8.81) |
| Cardiac disorders | Acute coronary syndrome | 10051592 | 13 | 25.79(14.96,44.46) | 25.75(308.78) | 4.68(2.44) | 25.71(14.91) |
| Investigations | Blood urine present | 10018870 | 13 | 9.53(5.53,16.43) | 9.52(99.04) | 3.25(1.79) | 9.51(5.52) |
| Cardiac disorders | Myocardial ischaemia | 10028600 | 13 | 16.50(9.57,28.44) | 16.47(188.76) | 4.04(2.19) | 16.46(9.55) |
| Cardiac disorders | Ventricular tachycardia | 10047302 | 13 | 9.44(5.48,16.28) | 9.43(97.90) | 3.24(1.78) | 9.42(5.47) |
| Vascular disorders | Embolism | 10061169 | 13 | 17.43(10.12,30.05) | 17.40(200.80) | 4.12(2.23) | 17.39(10.09) |
| Cardiac disorders | Coronary artery disease | 10011078 | 12 | 5.67(3.22,10.00) | 5.67(46.11) | 2.50(1.26) | 5.66(3.22) |
| Nervous system disorders | Cerebrovascular disorder | 10008196 | 11 | 10.16(5.62,18.36) | 10.14(90.64) | 3.34(1.69) | 10.14(5.61) |
| Investigations | Coagulation time prolonged | 10009799 | 11 | 23.27(12.87,42.05) | 23.23(233.73) | 4.54(2.19) | 23.20(12.84) |
| Cardiac disorders | Pericardial effusion | 10034474 | 11 | 6.48(3.59,11.71) | 6.47(50.89) | 2.69(1.32) | 6.47(3.58) |
| General disorders and administration site conditions | Extravasation | 10015866 | 11 | 21.04(11.64,38.03) | 21.01(209.41) | 4.39(2.14) | 20.99(11.61) |
| Injury, poisoning and procedural complications | Post procedural complication | 10058046 | 11 | 11.04(6.11,19.95) | 11.02(100.19) | 3.46(1.75) | 11.02(6.10) |
| Injury, poisoning and procedural complications | Labelled drug-drug interaction medication error | 10064373 | 10 | 14.91(8.01,27.72) | 14.89(129.43) | 3.89(1.85) | 14.87(8.00) |
| General disorders and administration site conditions | Vascular stent thrombosis | 10063934 | 10 | 98.24(52.74,182.99) | 98.10(955.71) | 6.61(2.45) | 97.55(52.37) |
| Injury, poisoning and procedural complications | Implantation complication | 10021547 | 10 | 178.57(95.73,333.09) | 178.31(1745.25) | 7.46(2.50) | 176.51(94.63) |
| Blood and lymphatic system disorders | Disseminated intravascular coagulation | 10013442 | 10 | 11.46(6.16,21.31) | 11.45(95.28) | 3.52(1.68) | 11.44(6.15) |
| Cardiac disorders | Coronary artery thrombosis | 10011091 | 10 | 57.61(30.95,107.24) | 57.53(553.65) | 5.84(2.36) | 57.34(30.81) |
| Nervous system disorders | Brain oedema | 10048962 | 10 | 11.45(6.16,21.30) | 11.44(95.21) | 3.51(1.68) | 11.43(6.15) |
| Cardiac disorders | Coronary artery stenosis | 10011089 | 10 | 37.80(20.31,70.33) | 37.74(356.92) | 5.24(2.25) | 37.66(20.24) |
| Respiratory, thoracic and mediastinal disorders | Respiratory tract haemorrhage | 10038727 | 9 | 153.45(79.58,295.91) | 153.25(1349.46) | 7.25(2.32) | 151.92(78.79) |
| Injury, poisoning and procedural complications | Procedural site reaction | 10036769 | 9 | 135.78(70.44,261.75) | 135.60(1193.22) | 7.07(2.31) | 134.56(69.81) |
| Blood and lymphatic system disorders | Immune thrombocytopenia | 10083842 | 9 | 9.43(4.91,18.14) | 9.42(67.73) | 3.24(1.44) | 9.42(4.90) |
| Renal and urinary disorders | Azotaemia | 10003885 | 8 | 4.88(2.44,9.76) | 4.87(24.63) | 2.28(0.81) | 4.87(2.44) |
| Eye disorders | Conjunctival haemorrhage | 10010719 | 8 | 15.76(7.87,31.53) | 15.74(110.34) | 3.98(1.61) | 15.73(7.86) |
| Investigations | Drug specific antibody present | 10013745 | 8 | 22.67(11.33,45.36) | 22.64(165.28) | 4.50(1.77) | 22.61(11.30) |
| Cardiac disorders | Angina unstable | 10002388 | 8 | 18.26(9.13,36.55) | 18.24(130.24) | 4.19(1.68) | 18.22(9.11) |
| Cardiac disorders | Cardiac ventricular thrombosis | 10053994 | 8 | 27.93(13.95,55.91) | 27.90(207.16) | 4.80(1.84) | 27.86(13.92) |
| Blood and lymphatic system disorders | Haemolysis | 10018910 | 7 | 10.96(5.22,23.01) | 10.95(63.26) | 3.45(1.27) | 10.94(5.21) |
| Gastrointestinal disorders | Haemoperitoneum | 10018935 | 7 | 38.29(18.23,80.41) | 38.25(253.37) | 5.25(1.73) | 38.17(18.17) |
| General disorders and administration site conditions | Sudden death | 10042434 | 7 | 6.85(3.27,14.38) | 6.85(34.95) | 2.78(0.96) | 6.85(3.26) |
| Surgical and medical procedures | Coronary artery bypass | 10011077 | 7 | 20.14(9.59,42.28) | 20.12(127.04) | 4.33(1.55) | 20.10(9.57) |
| Renal and urinary disorders | Haemorrhage urinary tract | 10055847 | 7 | 35.50(16.90,74.55) | 35.46(233.97) | 5.15(1.72) | 35.39(16.85) |
| General disorders and administration site conditions | Mucosal haemorrhage | 10061298 | 7 | 81.62(38.83,171.58) | 81.54(554.26) | 6.34(1.86) | 81.16(38.61) |
| Vascular disorders | Angiopathy | 10059245 | 6 | 11.37(5.11,25.33) | 11.36(56.67) | 3.51(1.10) | 11.36(5.10) |
| Investigations | Drug specific antibody | 10080179 | 6 | 29.66(13.31,66.08) | 29.63(165.70) | 4.89(1.45) | 29.58(13.28) |
| Investigations | Occult blood positive | 10061880 | 6 | 20.41(9.16,45.46) | 20.39(110.51) | 4.35(1.34) | 20.37(9.14) |
| Injury, poisoning and procedural complications | Labelled drug-drug interaction issue | 10084721 | 6 | 52.20(23.42,116.38) | 52.16(300.15) | 5.70(1.56) | 52.00(23.33) |
| Injury, poisoning and procedural complications | Brain herniation | 10006126 | 6 | 34.39(15.43,76.63) | 34.36(193.95) | 5.10(1.48) | 34.29(15.39) |
| Nervous system disorders | Intraventricular haemorrhage | 10022840 | 6 | 28.90(12.97,64.40) | 28.88(161.20) | 4.85(1.44) | 28.83(12.94) |
| Respiratory, thoracic and mediastinal disorders | Suffocation feeling | 10042444 | 6 | 5.76(2.59,12.83) | 5.76(23.58) | 2.52(0.69) | 5.75(2.58) |
| Cardiac disorders | Pericardial haemorrhage | 10034476 | 6 | 37.61(16.87,83.81) | 37.57(213.14) | 5.23(1.50) | 37.49(16.82) |
| Renal and urinary disorders | Anuria | 10002847 | 6 | 10.00(4.49,22.26) | 9.99(48.51) | 3.32(1.04) | 9.98(4.48) |
| Vascular disorders | Haemodynamic instability | 10052076 | 6 | 17.17(7.71,38.24) | 17.15(91.19) | 4.10(1.28) | 17.14(7.69) |
| Nervous system disorders | Haemorrhagic stroke | 10019016 | 5 | 7.53(3.13,18.09) | 7.52(28.26) | 2.91(0.67) | 7.52(3.13) |
| Respiratory, thoracic and mediastinal disorders | Pharyngeal haemorrhage | 10034827 | 5 | 56.25(23.37,135.38) | 56.21(270.27) | 5.81(1.28) | 56.03(23.28) |
| General disorders and administration site conditions | Catheter site haematoma | 10055662 | 5 | 237.27(98.14,573.64) | 237.09(1159.69) | 7.87(1.37) | 233.92(96.75) |
| Nervous system disorders | Hemiplegia | 10019468 | 5 | 7.04(2.93,16.92) | 7.03(25.87) | 2.81(0.63) | 7.03(2.93) |
| General disorders and administration site conditions | Injection site extravasation | 10022062 | 5 | 5.50(2.29,13.22) | 5.50(18.39) | 2.46(0.47) | 5.50(2.29) |
| Eye disorders | Eye haemorrhage | 10015926 | 5 | 4.84(2.01,11.63) | 4.84(15.21) | 2.27(0.38) | 4.83(2.01) |
| Blood and lymphatic system disorders | Thrombocytopenic purpura | 10043561 | 5 | 9.49(3.95,22.81) | 9.48(37.93) | 3.24(0.79) | 9.48(3.94) |
| Cardiac disorders | Pulseless electrical activity | 10058151 | 5 | 16.21(6.74,38.98) | 16.20(71.25) | 4.02(1.02) | 16.19(6.73) |
| Investigations | Procalcitonin increased | 10067081 | 4 | 42.03(15.75,112.16) | 42.01(159.75) | 5.39(0.90) | 41.91(15.71) |
| Blood and lymphatic system disorders | Blood loss anaemia | 10082297 | 4 | 6.95(2.61,18.54) | 6.95(20.37) | 2.80(0.37) | 6.95(2.61) |
| Blood and lymphatic system disorders | Blood disorder | 10061590 | 4 | 7.33(2.75,19.53) | 7.32(21.83) | 2.87(0.40) | 7.32(2.75) |
| Product issues | Thrombosis in device | 10062546 | 4 | 20.49(7.68,54.65) | 20.48(74.03) | 4.35(0.77) | 20.46(7.67) |
| Injury, poisoning and procedural complications | Vascular access site haemorrhage | 10077643 | 4 | 359.03(133.36,966.56) | 358.82(1398.45) | 8.46(1.00) | 351.59(130.60) |
| Gastrointestinal disorders | Intestinal haemorrhage | 10059175 | 4 | 10.11(3.79,26.95) | 10.10(32.79) | 3.34(0.55) | 10.10(3.79) |
| General disorders and administration site conditions | Vessel puncture site haematoma | 10065902 | 4 | 201.89(75.32,541.13) | 201.77(789.94) | 7.64(0.99) | 199.47(74.42) |
| Cardiac disorders | Myocardial rupture | 10028604 | 4 | 215.64(80.42,578.21) | 215.51(843.62) | 7.73(0.99) | 212.89(79.39) |
| Blood and lymphatic system disorders | Thrombotic microangiopathy | 10043645 | 4 | 7.59(2.85,20.23) | 7.58(22.85) | 2.92(0.42) | 7.58(2.84) |
| Injury, poisoning and procedural complications | Extradural haematoma | 10015769 | 4 | 34.88(13.07,93.05) | 34.86(131.28) | 5.12(0.87) | 34.79(13.04) |
| Nervous system disorders | Cerebral haematoma | 10053942 | 4 | 10.14(3.80,27.04) | 10.14(32.93) | 3.34(0.55) | 10.13(3.80) |
| Investigations | Activated partial thromboplastin time prolonged | 10000636 | 4 | 10.31(3.87,27.49) | 10.31(33.60) | 3.36(0.56) | 10.30(3.86) |
| Surgical and medical procedures | Angioplasty | 10002475 | 4 | 42.42(15.90,113.19) | 42.39(161.28) | 5.40(0.90) | 42.29(15.85) |
| Investigations | Bleeding time prolonged | 10005140 | 4 | 17.74(6.65,47.31) | 17.73(63.09) | 4.15(0.74) | 17.71(6.64) |
| Nervous system disorders | Cerebral artery occlusion | 10008089 | 4 | 44.39(16.64,118.46) | 44.37(169.13) | 5.47(0.90) | 44.26(16.58) |
| Nervous system disorders | Cerebellar haemorrhage | 10008030 | 4 | 22.06(8.27,58.84) | 22.05(80.28) | 4.46(0.79) | 22.02(8.26) |
| Nervous system disorders | Haemorrhagic transformation stroke | 10055677 | 4 | 32.85(12.32,87.64) | 32.84(123.23) | 5.03(0.86) | 32.78(12.29) |
| Vascular disorders | Hypovolaemic shock | 10021138 | 4 | 13.99(5.25,37.31) | 13.99(48.20) | 3.80(0.67) | 13.98(5.24) |
| Investigations | Electrocardiogram ST segment elevation | 10014392 | 4 | 13.92(5.22,37.13) | 13.92(47.92) | 3.80(0.67) | 13.91(5.22) |
| Nervous system disorders | Brain stem infarction | 10006147 | 3 | 32.79(10.56,101.80) | 32.77(92.24) | 5.03(0.43) | 32.71(10.54) |
| Injury, poisoning and procedural complications | Coronary artery restenosis | 10056489 | 3 | 79.14(25.45,246.07) | 79.10(230.30) | 6.30(0.50) | 78.75(25.33) |
| Respiratory, thoracic and mediastinal disorders | Acute pulmonary oedema | 10001029 | 3 | 9.64(3.11,29.90) | 9.63(23.20) | 3.27(0.16) | 9.63(3.10) |
| Gastrointestinal disorders | Duodenitis | 10013864 | 3 | 12.71(4.10,39.43) | 12.70(32.32) | 3.67(0.25) | 12.69(4.09) |
| Investigations | Protein urine present | 10053123 | 3 | 9.07(2.92,28.13) | 9.06(21.51) | 3.18(0.14) | 9.06(2.92) |
| Gastrointestinal disorders | Gastritis erosive | 10017865 | 3 | 8.79(2.83,27.25) | 8.78(20.68) | 3.13(0.13) | 8.78(2.83) |
| Nervous system disorders | Haemorrhagic cerebral infarction | 10019005 | 3 | 38.66(12.45,120.05) | 38.64(109.77) | 5.27(0.45) | 38.56(12.42) |
| Vascular disorders | Embolism arterial | 10014513 | 3 | 24.44(7.87,75.86) | 24.43(67.32) | 4.61(0.39) | 24.40(7.86) |
| Respiratory, thoracic and mediastinal disorders | Haemothorax | 10019027 | 3 | 13.89(4.48,43.11) | 13.89(35.85) | 3.79(0.27) | 13.88(4.47) |
| Vascular disorders | Arterial thrombosis | 10003178 | 3 | 16.07(5.18,49.85) | 16.06(42.33) | 4.00(0.31) | 16.05(5.17) |
| Investigations | Ejection fraction abnormal | 10014331 | 3 | 20.94(6.75,64.99) | 20.93(56.88) | 4.39(0.36) | 20.91(6.74) |
| Blood and lymphatic system disorders | Platelet disorder | 10035532 | 3 | 12.12(3.91,37.62) | 12.12(30.58) | 3.60(0.24) | 12.11(3.90) |
| Nervous system disorders | Spinal epidural haematoma | 10050162 | 3 | 69.09(22.23,214.75) | 69.06(200.42) | 6.10(0.49) | 68.79(22.13) |
| Nervous system disorders | Hydrocephalus | 10020508 | 3 | 8.51(2.74,26.40) | 8.51(19.86) | 3.09(0.12) | 8.50(2.74) |
| Injury, poisoning and procedural complications | Subcutaneous haematoma | 10042345 | 3 | 16.04(5.17,49.78) | 16.03(42.25) | 4.00(0.31) | 16.02(5.16) |
| Gastrointestinal disorders | Peptic ulcer haemorrhage | 10034344 | 3 | 27.71(8.93,86.01) | 27.70(77.08) | 4.79(0.41) | 27.65(8.91) |
| Investigations | Blood culture positive | 10005488 | 3 | 8.83(2.85,27.39) | 8.83(20.81) | 3.14(0.13) | 8.82(2.84) |
| Gastrointestinal disorders | Intra-abdominal haemorrhage | 10061249 | 3 | 15.08(4.86,46.79) | 15.07(39.38) | 3.91(0.29) | 15.06(4.85) |
| Gastrointestinal disorders | Retroperitoneal haematoma | 10058360 | 3 | 15.23(4.91,47.26) | 15.23(39.84) | 3.93(0.30) | 15.21(4.90) |
| Investigations | Klebsiella test positive | 10070091 | 3 | 67.39(21.68,209.48) | 67.37(195.38) | 6.07(0.49) | 67.11(21.59) |
| Injury, poisoning and procedural complications | Vascular access site haematoma | 10077647 | 3 | 1411.65(435.15,4579.49) | 1411.02(3910.04) | 10.35(0.48) | 1305.27(402.36) |
| Nervous system disorders | Basilar artery thrombosis | 10063093 | 3 | 178.26(57.15,556.07) | 178.18(523.21) | 7.46(0.52) | 176.39(56.55) |

**Table S8. Tirofiban-related AE signals at the PT level in both the FAERS and WHO-VigiAccess databases**

| **Preferred Term(PT)** | ***FAERS database*** | | | | | ***WHO-VigiAccess database*** | | | | |
| --- | --- | --- | --- | --- | --- | --- | --- | --- | --- | --- |
|  | **Case reports** | **ROR(95% CI)** | **PRR(Chi_Square)** | **IC(IC025)** | **EBGM(EBGM05)** | **Case reports** | **ROR(95% CI)** | **PRR(Chi_Square)** | **IC(IC025)** | **EBGM(EBGM05)** |
| Haemorrhage | 370 | 71.12(63.84,79.23) | 63.68(22774.5) | 5.99(5.60) | 63.43(56.94) | 223 | 28.48(24.92,32.55) | 27.58(5709.44) | 4.78(4.43) | 27.53(24.09) |
| Thrombocytopenia | 248 | 42.79(37.60,48.70) | 39.82(9379.40) | 5.31(4.91) | 39.72(34.91) | 1391 | 89.61(84.47,95.06) | 71.40(96442.6) | 6.15(6.00) | 71.11(67.04) |
| Myocardial infarction* | 207 | 20.95(18.20,24.11) | 19.76(3693.82) | 4.30(3.97) | 19.74(17.15) | 89 | 7.49(6.08,9.24) | 7.41(493.92) | 2.89(2.48) | 7.40(6.01) |
| Angina pectoris* | 181 | 113.77(97.91,132.19) | 107.91(19051.5) | 6.74(5.86) | 107.19(92.25) | 26 | 8.73(5.94,12.83) | 8.70(177.13) | 3.12(2.20) | 8.69(5.91) |
| Haemorrhage intracranial | 176 | 211.77(181.81,246.66) | 201.13(34618.0) | 7.63(6.33) | 198.63(170.53) | 77 | 56.01(44.73,70.14) | 55.39(4099.89) | 5.79(4.70) | 55.21(44.09) |
| Cerebral haemorrhage | 96 | 48.33(39.44,59.21) | 47.02(4314.07) | 5.55(4.69) | 46.89(38.27) | 105 | 29.47(24.30,35.74) | 29.03(2838.33) | 4.86(4.24) | 28.98(23.90) |
| Cardiac failure* | 80 | 17.95(14.38,22.41) | 17.56(1249.56) | 4.13(3.54) | 17.54(14.05) | 25 | 4.72(3.19,6.99) | 4.71(73.02) | 2.23(1.47) | 4.71(3.18) |
| Vascular stent thrombosis* | 55 | 502.58(383.49,658.66) | 494.67(26278.9) | 8.91(5.26) | 479.75(366.07) | 10 | 98.24(52.74,182.99) | 98.10(955.71) | 6.61(2.45) | 97.55(52.37) |
| Cardiogenic shock* | 38 | 49.84(36.18,68.64) | 49.30(1793.13) | 5.62(3.99) | 49.15(35.69) | 30 | 37.88(26.45,54.24) | 37.72(1070.11) | 5.23(3.59) | 37.64(26.28) |
| Haemoglobin decreased | 32 | 5.43(3.84,7.70) | 5.39(114.64) | 2.43(1.75) | 5.39(3.81) | 80 | 10.12(8.12,12.62) | 10.01(649.45) | 3.32(2.85) | 10.01(8.03) |
| Cardiac death* | 31 | 453.85(317.10,649.56) | 449.82(13500.3) | 8.77(4.38) | 437.46(305.65) | 20 | 288.73(185.49,449.45) | 287.88(5624.77) | 8.15(3.66) | 283.22(181.94) |
| Gastrointestinal haemorrhage | 29 | 5.89(4.09,8.49) | 5.85(116.69) | 2.55(1.80) | 5.85(4.06) | 138 | 16.28(13.76,19.28) | 15.97(1937.50) | 4.00(3.60) | 15.96(13.48) |
| Acute myocardial infarction* | 25 | 14.43(9.73,21.38) | 14.33(309.89) | 3.84(2.67) | 14.32(9.66) | 17 | 8.68(5.39,13.97) | 8.66(115.11) | 3.11(1.92) | 8.65(5.38) |
| Coronary artery stenosis* | 24 | 80.28(53.68,120.06) | 79.74(1856.83) | 6.31(3.68) | 79.34(53.05) | 10 | 37.80(20.31,70.33) | 37.74(356.92) | 5.24(2.25) | 37.66(20.24) |
| Haematoma | 24 | 15.94(10.66,23.81) | 15.83(333.32) | 3.98(2.73) | 15.82(10.59) | 147 | 40.25(34.18,47.41) | 39.40(5491.92) | 5.30(4.72) | 39.31(33.38) |
| Haematuria | 23 | 11.62(7.71,17.51) | 11.55(221.57) | 3.53(2.41) | 11.54(7.66) | 190 | 44.02(38.10,50.86) | 42.81(7745.08) | 5.42(4.92) | 42.71(36.97) |
| Haemoptysis | 20 | 12.55(8.09,19.49) | 12.49(211.30) | 3.64(2.38) | 12.48(8.04) | 88 | 36.81(29.82,45.43) | 36.34(3019.10) | 5.18(4.39) | 36.27(29.38) |
| Pulmonary alveolar haemorrhage | 19 | 62.68(39.90,98.47) | 62.34(1142.43) | 5.96(3.29) | 62.10(39.53) | 20 | 62.36(40.17,96.79) | 62.17(1199.58) | 5.95(3.36) | 61.96(39.91) |
| Thrombosis* | 18 | 3.91(2.46,6.21) | 3.90(38.78) | 1.96(1.09) | 3.89(2.45) | 25 | 4.23(2.86,6.26) | 4.22(61.39) | 2.08(1.34) | 4.22(2.85) |
| Thrombosis in device* | 18 | 90.34(56.78,143.75) | 89.88(1573.28) | 6.48(3.32) | 89.38(56.17) | 4 | 20.49(7.68,54.65) | 20.48(74.03) | 4.35(0.77) | 20.46(7.67) |
| Embolism* | 17 | 35.39(21.97,57.03) | 35.23(564.15) | 5.14(2.92) | 35.15(21.81) | 13 | 17.43(10.12,30.05) | 17.40(200.80) | 4.12(2.23) | 17.39(10.09) |
| Catheter site haemorrhage | 15 | 110.15(66.22,183.23) | 109.68(1604.32) | 6.77(3.09) | 108.93(65.49) | 15 | 92.80(55.84,154.23) | 92.60(1352.00) | 6.53(3.06) | 92.12(55.43) |
| Petechiae | 14 | 23.96(14.17,40.51) | 23.86(306.27) | 4.57(2.49) | 23.83(14.09) | 37 | 17.75(12.85,24.52) | 17.66(581.07) | 4.14(3.15) | 17.64(12.77) |
| Ventricular fibrillation* | 14 | 22.04(13.04,37.27) | 21.96(279.72) | 4.45(2.45) | 21.93(12.97) | 17 | 17.56(10.91,28.28) | 17.52(264.60) | 4.13(2.51) | 17.50(10.87) |
| Post procedural complication | 14 | 12.61(7.46,21.31) | 12.56(148.90) | 3.65(2.08) | 12.55(7.42) | 11 | 11.04(6.11,19.95) | 11.02(100.19) | 3.46(1.75) | 11.02(6.10) |
| Gingival bleeding | 13 | 16.78(9.73,28.94) | 16.72(192.02) | 4.06(2.20) | 16.71(9.69) | 157 | 105.86(90.32,124.07) | 103.43(15834.9) | 6.68(5.73) | 102.82(87.73) |
| Retroperitoneal haemorrhage | 13 | 88.48(51.25,152.76) | 88.15(1114.00) | 6.45(2.83) | 87.67(50.78) | 40 | 129.26(94.62,176.58) | 128.50(5023.60) | 7.00(4.51) | 127.57(93.38) |
| Haematocrit decreased | 11 | 9.37(5.18,16.93) | 9.34(81.91) | 3.22(1.63) | 9.34(5.16) | 32 | 19.31(13.64,27.33) | 19.22(552.24) | 4.26(3.13) | 19.20(13.56) |
| Drug specific antibody present* | 10 | 27.71(14.89,51.58) | 27.64(256.31) | 4.79(2.14) | 27.59(14.82) | 8 | 22.67(11.33,45.36) | 22.64(165.28) | 4.50(1.77) | 22.61(11.30) |
| Pulmonary haemorrhage | 10 | 21.86(11.74,40.67) | 21.80(198.18) | 4.44(2.04) | 21.77(11.70) | 50 | 81.10(61.36,107.18) | 80.51(3908.31) | 6.32(4.57) | 80.14(60.64) |
| Upper gastrointestinal haemorrhage | 10 | 10.25(5.51,19.07) | 10.22(83.19) | 3.35(1.60) | 10.22(5.49) | 57 | 30.77(23.70,39.94) | 30.52(1624.98) | 4.93(3.96) | 30.47(23.47) |
| Acute coronary syndrome | 10 | 20.37(10.95,37.91) | 20.32(183.46) | 4.34(2.01) | 20.29(10.90) | 13 | 25.79(14.96,44.46) | 25.75(308.78) | 4.68(2.44) | 25.71(14.91) |
| Haematemesis | 9 | 6.17(3.21,11.86) | 6.15(38.84) | 2.62(1.11) | 6.15(3.20) | 71 | 25.69(20.33,32.46) | 25.43(1664.53) | 4.67(3.90) | 25.39(20.10) |
| Subarachnoid haemorrhage | 9 | 15.35(7.98,29.53) | 15.31(120.28) | 3.94(1.74) | 15.30(7.95) | 24 | 27.62(18.49,41.25) | 27.52(612.54) | 4.78(3.16) | 27.48(18.40) |
| Shock haemorrhagic | 9 | 20.19(10.49,38.85) | 20.14(163.52) | 4.33(1.87) | 20.12(10.45) | 17 | 28.16(17.49,45.34) | 28.09(443.51) | 4.81(2.80) | 28.05(17.42) |
| Post procedural haemorrhage | 9 | 13.35(6.94,25.69) | 13.32(102.50) | 3.73(1.66) | 13.31(6.92) | 18 | 20.20(12.72,32.09) | 20.15(327.28) | 4.33(2.66) | 20.13(12.67) |
| Cardiac ventricular thrombosis* | 9 | 36.09(18.75,69.47) | 36.00(305.55) | 5.17(2.08) | 35.92(18.66) | 8 | 27.93(13.95,55.91) | 27.90(207.16) | 4.80(1.84) | 27.86(13.92) |
| Coronary artery thrombosis* | 8 | 66.54(33.20,133.35) | 66.39(513.08) | 6.05(2.04) | 66.11(32.99) | 10 | 57.61(30.95,107.24) | 57.53(553.65) | 5.84(2.36) | 57.34(30.81) |
| Ventricular tachycardia | 8 | 8.50(4.25,17.02) | 8.49(52.82) | 3.08(1.25) | 8.48(4.24) | 13 | 9.44(5.48,16.28) | 9.43(97.90) | 3.24(1.78) | 9.42(5.47) |
| Pulseless electrical activity* | 8 | 28.69(14.32,57.44) | 28.62(212.89) | 4.84(1.85) | 28.57(14.27) | 5 | 16.21(6.74,38.98) | 16.20(71.25) | 4.02(1.02) | 16.19(6.73) |
| Haemolysis | 7 | 15.99(7.61,33.57) | 15.96(98.05) | 3.99(1.45) | 15.94(7.59) | 7 | 10.96(5.22,23.01) | 10.95(63.26) | 3.45(1.27) | 10.94(5.21) |
| Cardiac tamponade* | 6 | 22.09(9.91,49.24) | 22.06(120.46) | 4.46(1.37) | 22.03(9.88) | 17 | 60.62(37.63,97.66) | 60.48(990.96) | 5.91(3.13) | 60.27(37.41) |
| Coronary artery bypass | 6 | 17.02(7.64,37.92) | 16.99(90.21) | 4.09(1.28) | 16.97(7.62) | 7 | 20.14(9.59,42.28) | 20.12(127.04) | 4.33(1.55) | 20.10(9.57) |
| Haemodynamic instability | 6 | 14.68(6.59,32.71) | 14.66(76.28) | 3.87(1.22) | 14.64(6.57) | 6 | 17.17(7.71,38.24) | 17.15(91.19) | 4.10(1.28) | 17.14(7.69) |
| Vascular access site haemorrhage | 6 | 883.43(388.02,2011.38) | 881.91(5001.61) | 9.71(1.67) | 835.55(366.99) | 4 | 359.03(133.36,966.56) | 358.82(1398.45) | 8.46(1.00) | 351.59(130.60) |
| Activated partial thromboplastin time prolonged | 5 | 16.59(6.90,39.91) | 16.57(73.08) | 4.05(1.02) | 16.55(6.88) | 4 | 10.31(3.87,27.49) | 10.31(33.60) | 3.36(0.56) | 10.30(3.86) |
| Acute pulmonary oedema | 5 | 15.47(6.43,37.22) | 15.45(67.53) | 3.95(1.00) | 15.44(6.42) | 3 | 9.64(3.11,29.90) | 9.63(23.20) | 3.27(0.16) | 9.63(3.10) |
| Coagulopathy | 5 | 5.23(2.17,12.57) | 5.22(17.06) | 2.38(0.44) | 5.22(2.17) | 26 | 13.73(9.34,20.18) | 13.68(305.37) | 3.77(2.66) | 13.67(9.30) |
| Disseminated intravascular coagulation | 5 | 6.10(2.54,14.66) | 6.09(21.26) | 2.61(0.54) | 6.09(2.53) | 10 | 11.46(6.16,21.31) | 11.45(95.28) | 3.52(1.68) | 11.44(6.15) |
| Ecchymosis | 5 | 12.50(5.20,30.07) | 12.49(52.80) | 3.64(0.92) | 12.48(5.19) | 39 | 29.92(21.84,41.00) | 29.76(1082.15) | 4.89(3.65) | 29.71(21.68) |
| Haemorrhagic stroke | 5 | 11.02(4.58,26.50) | 11.00(45.45) | 3.46(0.86) | 11.00(4.57) | 5 | 7.53(3.13,18.09) | 7.52(28.26) | 2.91(0.67) | 7.52(3.13) |
| Catheter site haematoma | 5 | 308.08(127.07,746.94) | 307.64(1499.19) | 8.24(1.37) | 301.81(124.49) | 5 | 237.27(98.14,573.64) | 237.09(1159.69) | 7.87(1.37) | 233.92(96.75) |
| Angina unstable | 4 | 9.95(3.73,26.52) | 9.94(32.13) | 3.31(0.54) | 9.93(3.72) | 8 | 18.26(9.13,36.55) | 18.24(130.24) | 4.19(1.68) | 18.22(9.11) |
| Brain herniation* | 4 | 23.92(8.96,63.80) | 23.89(87.60) | 4.58(0.81) | 23.85(8.94) | 6 | 34.39(15.43,76.63) | 34.36(193.95) | 5.10(1.48) | 34.29(15.39) |
| Electrocardiogram ST segment elevation* | 4 | 20.68(7.75,55.16) | 20.66(74.73) | 4.37(0.77) | 20.63(7.73) | 4 | 13.92(5.22,37.13) | 13.92(47.92) | 3.80(0.67) | 13.91(5.22) |
| Hypovolaemic shock | 4 | 15.01(5.63,40.04) | 15.00(52.21) | 3.91(0.69) | 14.98(5.62) | 4 | 13.99(5.25,37.31) | 13.99(48.20) | 3.80(0.67) | 13.98(5.24) |
| Brain oedema | 4 | 5.66(2.12,15.08) | 5.65(15.31) | 2.50(0.26) | 5.65(2.12) | 10 | 11.45(6.16,21.30) | 11.44(95.21) | 3.51(1.68) | 11.43(6.15) |
| Coronary artery restenosis* | 4 | 106.84(39.94,285.77) | 106.72(416.12) | 6.73(0.97) | 106.01(39.63) | 3 | 79.14(25.45,246.07) | 79.10(230.30) | 6.30(0.50) | 78.75(25.33) |
| Brain stem infarction* | 3 | 47.95(15.43,149.00) | 47.91(137.38) | 5.58(0.47) | 47.77(15.37) | 3 | 32.79(10.56,101.80) | 32.77(92.24) | 5.03(0.43) | 32.71(10.54) |
| Haemothorax | 3 | 17.01(5.48,52.80) | 17.00(45.12) | 4.09(0.32) | 16.98(5.47) | 3 | 13.89(4.48,43.11) | 13.89(35.85) | 3.79(0.27) | 13.88(4.47) |
| Intraventricular haemorrhage | 3 | 20.38(6.56,63.26) | 20.36(55.16) | 4.35(0.36) | 20.34(6.55) | 6 | 28.90(12.97,64.40) | 28.88(161.20) | 4.85(1.44) | 28.83(12.94) |
| Retroperitoneal haematoma | 3 | 23.65(7.62,73.44) | 23.63(64.94) | 4.56(0.38) | 23.60(7.60) | 3 | 15.23(4.91,47.26) | 15.23(39.84) | 3.93(0.30) | 15.21(4.90) |
| Mucosal haemorrhage | 3 | 45.44(14.62,141.18) | 45.40(129.89) | 5.50(0.46) | 45.27(14.57) | 7 | 81.62(38.83,171.58) | 81.54(554.26) | 6.34(1.86) | 81.16(38.61) |

*: Not mentioned in the drug label.

**Table S9. Distribution and ranking of Preferred Term–level AE reports in the FAERS database stratified by healthcare professional reporter type.**

| **System Organ Class (SOC)** | **Preferred Term (PT)** | **PT code** | **Case reports** | **ROR (95% CI)** | **PRR(χ2)** | **IC(IC025)** | **EBGM(EBGM05)** |
| --- | --- | --- | --- | --- | --- | --- | --- |
| Vascular disorders | Haemorrhage | 10055798 | 367 | 79.19(70.99,88.34) | 69.98(24790.8) | 6.12(5.71) | 69.41(62.22) |
| Blood and lymphatic system disorders | Thrombocytopenia | 10043554 | 220 | 24.27(21.16,27.84) | 22.63(4549.62) | 4.50(4.16) | 22.57(19.67) |
| Cardiac disorders | Myocardial infarction | 10028596 | 204 | 28.06(24.34,32.35) | 26.29(4959.11) | 4.71(4.34) | 26.21(22.73) |
| Cardiac disorders | Angina pectoris | 10002383 | 179 | 113.36(97.39,131.94) | 106.90(18555.0) | 6.72(5.84) | 105.58(90.71) |
| Nervous system disorders | Haemorrhage intracranial | 10018985 | 175 | 144.42(123.85,168.40) | 136.35(23152.0) | 7.07(6.03) | 134.22(115.10) |
| General disorders and administration site conditions | Death | 10011906 | 132 | 3.14(2.63,3.73) | 3.05(183.91) | 1.61(1.33) | 3.05(2.56) |
| Cardiac disorders | Cardiac failure acute | 10007556 | 116 | 215.76(178.85,260.30) | 207.76(23303.9) | 7.66(5.94) | 202.83(168.13) |
| Respiratory, thoracic and mediastinal disorders | Acute respiratory failure | 10001053 | 94 | 65.38(53.21,80.34) | 63.44(5736.50) | 5.98(4.95) | 62.97(51.25) |
| Nervous system disorders | Cerebral haemorrhage | 10008111 | 93 | 38.39(31.22,47.21) | 37.27(3271.36) | 5.21(4.44) | 37.12(30.18) |
| Cardiac disorders | Cardiac failure | 10007554 | 77 | 14.12(11.26,17.71) | 13.80(914.28) | 3.78(3.23) | 13.78(10.99) |
| Vascular disorders | Infarction | 10061216 | 53 | 193.53(147.07,254.66) | 190.25(9760.15) | 7.54(4.99) | 186.11(141.43) |
| General disorders and administration site conditions | Vascular stent thrombosis | 10063934 | 53 | 324.25(245.92,427.53) | 318.74(16182.0) | 8.26(5.12) | 307.27(233.04) |
| Cardiac disorders | Cardiogenic shock | 10007625 | 32 | 26.60(18.77,37.71) | 26.34(778.01) | 4.71(3.39) | 26.26(18.53) |
| General disorders and administration site conditions | Cardiac death | 10049993 | 31 | 333.25(232.38,477.90) | 329.94(9786.76) | 8.31(4.34) | 317.65(221.50) |
| Cardiac disorders | Arrhythmia | 10003119 | 27 | 10.38(7.10,15.16) | 10.29(226.50) | 3.36(2.40) | 10.28(7.04) |
| Gastrointestinal disorders | Gastrointestinal haemorrhage | 10017955 | 27 | 5.08(3.48,7.43) | 5.05(87.77) | 2.34(1.59) | 5.05(3.45) |
| Investigations | Haemoglobin decreased | 10018884 | 25 | 3.81(2.57,5.65) | 3.79(51.37) | 1.92(1.21) | 3.79(2.55) |
| Cardiac disorders | Acute myocardial infarction | 10000891 | 23 | 9.32(6.19,14.06) | 9.26(169.48) | 3.21(2.19) | 9.25(6.14) |
| Cardiac disorders | Coronary artery stenosis | 10011089 | 21 | 52.08(33.86,80.09) | 51.73(1038.61) | 5.68(3.35) | 51.43(33.44) |
| Renal and urinary disorders | Haematuria | 10018867 | 21 | 8.62(5.61,13.24) | 8.57(140.38) | 3.10(2.05) | 8.56(5.57) |
| Surgical and medical procedures | Craniectomy | 10052937 | 21 | 5993.13(3427.53,10479.2) | 5952.71(73508.9) | 11.77(3.71) | 3502.01(2002.83) |
| Vascular disorders | Haematoma | 10018852 | 20 | 11.13(7.17,17.27) | 11.06(182.89) | 3.47(2.27) | 11.05(7.12) |
| Surgical and medical procedures | Coronary revascularisation | 10049887 | 20 | 1860.62(1145.75,3021.51) | 1848.67(30338.3) | 10.57(3.69) | 1518.73(935.22) |
| Respiratory, thoracic and mediastinal disorders | Haemoptysis | 10018964 | 17 | 9.94(6.17,16.02) | 9.89(135.85) | 3.31(2.04) | 9.88(6.14) |
| Injury, poisoning and procedural complications | Reocclusion | 10038563 | 17 | 3159.99(1809.65,5517.91) | 3142.74(38985.1) | 11.16(3.39) | 2294.97(1314.28) |
| Cardiac disorders | Ventricular arrhythmia | 10047281 | 16 | 50.04(30.57,81.90) | 49.78(760.51) | 5.63(2.98) | 49.50(30.24) |
| Respiratory, thoracic and mediastinal disorders | Pulmonary alveolar haemorrhage | 10037313 | 13 | 26.49(15.35,45.71) | 26.38(316.56) | 4.72(2.45) | 26.31(15.24) |
| Injury, poisoning and procedural complications | Post procedural complication | 10058046 | 13 | 12.92(7.49,22.29) | 12.87(142.20) | 3.68(2.03) | 12.86(7.45) |
| Skin and subcutaneous tissue disorders | Petechiae | 10034754 | 12 | 16.71(9.47,29.47) | 16.65(176.18) | 4.05(2.11) | 16.62(9.42) |
| Cardiac disorders | Ventricular fibrillation | 10047290 | 12 | 12.64(7.17,22.29) | 12.59(127.93) | 3.65(1.93) | 12.58(7.13) |
| Gastrointestinal disorders | Gingival bleeding | 10018276 | 11 | 14.47(8.00,26.17) | 14.42(137.23) | 3.85(1.93) | 14.40(7.96) |
| Vascular disorders | Embolism | 10061169 | 11 | 17.50(9.67,31.65) | 17.44(170.15) | 4.12(2.04) | 17.41(9.62) |
| Product issues | Thrombosis in device | 10062546 | 11 | 40.39(22.31,73.11) | 40.25(419.08) | 5.32(2.40) | 40.07(22.13) |
| Injury, poisoning and procedural complications | Restenosis | 10082493 | 11 | 3476.82(1723.15,7015.24) | 3464.54(27062.5) | 11.27(2.62) | 2461.94(1220.16) |
| Investigations | Haematocrit decreased | 10018838 | 9 | 6.10(3.17,11.74) | 6.08(38.23) | 2.60(1.10) | 6.08(3.16) |
| Nervous system disorders | Subarachnoid haemorrhage | 10042316 | 9 | 10.67(5.54,20.53) | 10.64(78.53) | 3.41(1.52) | 10.63(5.52) |
| Gastrointestinal disorders | Upper gastrointestinal haemorrhage | 10046274 | 9 | 7.75(4.03,14.91) | 7.73(52.70) | 2.95(1.29) | 7.72(4.01) |
| General disorders and administration site conditions | Sudden cardiac death | 10049418 | 9 | 35.95(18.66,69.25) | 35.85(303.63) | 5.16(2.08) | 35.70(18.53) |
| Cardiac disorders | Acute coronary syndrome | 10051592 | 9 | 12.22(6.35,23.51) | 12.19(92.29) | 3.61(1.61) | 12.17(6.32) |
| Nervous system disorders | Intracranial haematoma | 10059491 | 9 | 206.89(106.70,401.15) | 206.29(1795.16) | 7.65(2.33) | 201.43(103.89) |
| Nervous system disorders | Ischaemic stroke | 10061256 | 9 | 6.24(3.24,12.01) | 6.22(39.45) | 2.64(1.12) | 6.22(3.23) |
| Investigations | Drug specific antibody present | 10013745 | 8 | 19.42(9.70,38.91) | 19.38(139.13) | 4.27(1.71) | 19.33(9.65) |
| Gastrointestinal disorders | Haematemesis | 10018830 | 8 | 4.74(2.37,9.48) | 4.73(23.51) | 2.24(0.78) | 4.73(2.36) |
| Gastrointestinal disorders | Retroperitoneal haemorrhage | 10038980 | 8 | 39.29(19.60,78.75) | 39.19(296.36) | 5.29(1.93) | 39.01(19.46) |
| Investigations | Ejection fraction decreased | 10050528 | 8 | 7.04(3.52,14.09) | 7.02(41.30) | 2.81(1.11) | 7.02(3.51) |
| Blood and lymphatic system disorders | Haemolysis | 10018910 | 7 | 12.69(6.04,26.66) | 12.67(75.13) | 3.66(1.34) | 12.65(6.02) |
| Respiratory, thoracic and mediastinal disorders | Pulmonary haemorrhage | 10037394 | 7 | 11.47(5.46,24.09) | 11.45(66.65) | 3.51(1.29) | 11.43(5.44) |
| Cardiac disorders | Ventricular tachycardia | 10047302 | 7 | 5.12(2.44,10.75) | 5.11(23.14) | 2.35(0.73) | 5.11(2.43) |
| Injury, poisoning and procedural complications | Post procedural haemorrhage | 10051077 | 7 | 10.85(5.17,22.79) | 10.83(62.38) | 3.44(1.26) | 10.82(5.15) |
| Surgical and medical procedures | Coronary artery bypass | 10011077 | 6 | 20.13(9.03,44.89) | 20.10(108.63) | 4.33(1.34) | 20.05(8.99) |
| Vascular disorders | Shock haemorrhagic | 10049771 | 6 | 9.07(4.07,20.21) | 9.05(42.94) | 3.18(0.98) | 9.04(4.06) |
| Cardiac disorders | Cardiac ventricular thrombosis | 10053994 | 6 | 21.49(9.64,47.91) | 21.45(116.68) | 4.42(1.36) | 21.40(9.60) |
| Cardiac disorders | Pulseless electrical activity | 10058151 | 6 | 13.87(6.22,30.93) | 13.85(71.43) | 3.79(1.19) | 13.83(6.20) |
| Cardiac disorders | Coronary artery thrombosis | 10011091 | 5 | 32.81(13.62,79.01) | 32.76(153.36) | 5.03(1.20) | 32.64(13.55) |
| Skin and subcutaneous tissue disorders | Ecchymosis | 10014080 | 5 | 9.89(4.11,23.80) | 9.88(39.86) | 3.30(0.81) | 9.87(4.10) |
| General disorders and administration site conditions | Catheter site haemorrhage | 10051099 | 5 | 31.73(13.18,76.42) | 31.68(148.03) | 4.98(1.19) | 31.57(13.11) |
| Investigations | Troponin T increased | 10058269 | 5 | 44.09(18.29,106.23) | 44.02(209.12) | 5.45(1.25) | 43.79(18.17) |
| Investigations | Activated partial thromboplastin time prolonged | 10000636 | 4 | 8.80(3.30,23.46) | 8.78(27.57) | 3.13(0.49) | 8.78(3.29) |
| Cardiac disorders | Angina unstable | 10002388 | 4 | 7.39(2.77,19.70) | 7.38(22.04) | 2.88(0.40) | 7.37(2.76) |
| Cardiac disorders | Bundle branch block left | 10006580 | 4 | 11.70(4.39,31.21) | 11.69(39.03) | 3.54(0.60) | 11.67(4.37) |
| Cardiac disorders | Cardiac tamponade | 10007610 | 4 | 9.51(3.56,25.36) | 9.50(30.38) | 3.25(0.52) | 9.49(3.56) |
| Nervous system disorders | Carotid artery stenosis | 10007687 | 4 | 15.04(5.64,40.14) | 15.02(52.28) | 3.91(0.69) | 15.00(5.62) |
| Investigations | Electrocardiogram ST segment elevation | 10014392 | 4 | 13.34(5.00,35.59) | 13.32(45.53) | 3.73(0.65) | 13.30(4.99) |
| Vascular disorders | Haemodynamic instability | 10052076 | 4 | 6.14(2.30,16.36) | 6.13(17.16) | 2.61(0.30) | 6.13(2.30) |
| Injury, poisoning and procedural complications | Coronary artery restenosis | 10056489 | 4 | 74.69(27.89,199.99) | 74.60(287.91) | 6.21(0.95) | 73.96(27.62) |
| Nervous system disorders | Capsular warning syndrome | 10067744 | 4 | 5676.54(1601.08,20125.9) | 5669.25(13601.4) | 11.73(0.76) | 3401.95(959.53) |
| General disorders and administration site conditions | Device embolisation | 10074896 | 4 | 188.17(69.82,507.12) | 187.93(727.67) | 7.52(0.98) | 183.89(68.23) |
| General disorders and administration site conditions | Vascular stent occlusion | 10077143 | 4 | 272.47(100.62,737.83) | 272.12(1047.01) | 8.04(0.98) | 263.72(97.39) |
| Injury, poisoning and procedural complications | Vascular access site haemorrhage | 10077643 | 4 | 431.13(157.79,1177.94) | 430.58(1631.70) | 8.68(0.98) | 409.87(150.01) |
| Injury, poisoning and procedural complications | Brain herniation | 10006126 | 3 | 11.76(3.79,36.50) | 11.75(29.45) | 3.55(0.23) | 11.73(3.78) |
| Respiratory, thoracic and mediastinal disorders | Haemothorax | 10019027 | 3 | 12.46(4.01,38.69) | 12.45(31.55) | 3.64(0.24) | 12.43(4.00) |
| Nervous system disorders | Intraventricular haemorrhage | 10022840 | 3 | 13.77(4.44,42.77) | 13.76(35.44) | 3.78(0.27) | 13.74(4.42) |
| Vascular disorders | Vascular rupture | 10053649 | 3 | 52.54(16.88,163.57) | 52.49(150.61) | 5.71(0.47) | 52.18(16.76) |
| General disorders and administration site conditions | Catheter site haematoma | 10055662 | 3 | 145.09(46.32,454.46) | 144.95(421.69) | 7.16(0.51) | 142.54(45.51) |
| Nervous system disorders | Embolic cerebral infarction | 10060839 | 3 | 50.17(16.12,156.16) | 50.12(143.58) | 5.64(0.47) | 49.83(16.01) |
| General disorders and administration site conditions | Disease complication | 10067671 | 3 | 10.83(3.49,33.63) | 10.82(26.72) | 3.43(0.20) | 10.81(3.48) |

**Table S10.Gender differences in specific PTs of tirofiban in FAERS database**

| **Preferred Term (PT)** | **Female**  **(case)** | **Male**  **(case)** | **ROR (95% CI)** | **χ²** | **P*** |
| --- | --- | --- | --- | --- | --- |
| **Thrombocytopenia** | **40** | **116** | **0.47(0.33,0.69)** | **15.7875** | **<.0001** |
| Myocardial infarction | 22 | 31 | 1.04(0.60,1.82) | 0.0236 | 0.8779 |
| Haemorrhage | 23 | 22 | 1.56(0.86,2.82) | 2.1662 | 0.1411 |
| **Angina pectoris** | **27** | **15** | **2.72(1.43,5.15)** | **10.1301** | **0.0015** |
| Cardiogenic shock | 14 | 23 | 0.89(0.46,1.75) | 0.1106 | 0.7395 |
| Haemoglobin decreased | 13 | 19 | 1.01(0.49,2.05) | 0.0002 | 0.9875 |
| Cardiac failure | 15 | 11 | 2.03(0.93,4.44) | 3.2445 | 0.0717 |
| Cerebral haemorrhage | 10 | 15 | 0.98(0.44,2.19) | 0.0025 | 0.9598 |
| **Coronary artery stenosis** | **17** | **7** | **3.64(1.50,8.82)** | **9.3071** | **0.0023** |
| Acute myocardial infarction | 11 | 13 | 1.25(0.56,2.80) | 0.2885 | 0.5912 |
| Haematoma | 8 | 10 | 1.18(0.46,3.00) | 0.1181 | 0.7311 |
| Pulmonary alveolar haemorrhage | 10 | 7 | 2.12(0.80,5.59) | 2.3962 | 0.1216 |
| Haemoptysis | 6 | 10 | 0.88(0.32,2.44) | 0.0600 | 0.8065 |
| Hypotension | 8 | 8 | 1.48(0.55,3.95) | 0.6064 | 0.4362 |
| Platelet count decreased | 7 | 8 | 1.29(0.47,3.57) | 0.2396 | 0.6245 |
| Ventricular fibrillation | 4 | 10 | 0.59(0.18,1.87) | 0.8326 | 0.3615 |
| Death | 5 | 9 | 0.82(0.27,2.44) | 0.1337 | 0.7146 |
| Post procedural complication | 4 | 10 | 0.59(0.18,1.87) | 0.8326 | 0.3615 |
| Thrombosis in device | 7 | 7 | 1.47(0.51,4.22) | 0.5299 | 0.4666 |
| Cardiac arrest | 7 | 6 | 1.72(0.58,5.15) | 0.9700 | 0.3247 |
| Petechiae | 6 | 7 | 1.26(0.42,3.77) | 0.1744 | 0.6762 |
| **Chest pain** | **9** | **3** | **4.46(1.20,16.52)** | **5.9747** | **0.0316** |
| **Anaemia** | **9** | **2** | **6.69(1.44,31.07)** | **7.8500** | **0.0126** |
| Gastrointestinal haemorrhage | 4 | 7 | 0.84(0.24,2.88) | 0.0783 | 1.0000 |
| Vascular stent thrombosis | 8 | 3 | 3.96(1.05,14.96) | 4.7763 | 0.0605 |
| Pyrexia | 4 | 7 | 0.84(0.24,2.88) | 0.0783 | 1.0000 |
| Haematocrit decreased | 5 | 6 | 1.23(0.37,4.04) | 0.1132 | 0.9774 |
| Haematuria | 1 | 10 | 0.15(0.02,1.14) | 4.5316 | 0.0687 |
| Acute coronary syndrome | 2 | 8 | 0.37(0.08,1.73) | 1.7533 | 0.3168 |

Abbreviations: FAERS, the U.S. Food and Drug Administration's Adverse Event Reporting System; PT, preferred term; ROR, reporting odds ratio; Cl, confidence interval.χ²:chi-square.

*:Chi-square or Fisher's exact tests.
